# Telesonography in emergency medicine: A systematic review

**DOI:** 10.1371/journal.pone.0194840

**Published:** 2018-05-03

**Authors:** Genevieve Marsh-Feiley, Leila Eadie, Philip Wilson

**Affiliations:** Centre for Rural Health, University of Aberdeen, Inverness, Scotland, United Kingdom; Universitat Regensburg, GERMANY

## Abstract

Ultrasound is an efficacious, versatile and affordable imaging technique in emergencies, but has limited utility without expert interpretation. Telesonography, in which experts may remotely support the use of ultrasound through a telecommunications link, may broaden access to ultrasound and improve patient outcomes, particularly in remote settings. This review assesses the literature regarding telesonography in emergency medicine, focussing on evidence of feasibility, diagnostic accuracy and clinical utility. A systematic search was performed for articles published from 1946 to February 2017 using the Cochrane, Medline, EMBASE, and CINAHL databases. Further searches utilising Scopus, Google Scholar, and citation lists were conducted. 4388 titles were identified and screened against inclusion criteria which resulted in the inclusion of 28 papers. These included feasibility, diagnostic accuracy and clinical pilot studies. Study design, methodology and quality were heterogeneous. There was good evidence of feasibility from multiple studies. Where sufficient bandwidth and high quality components were used, diagnostic accuracy was slightly reduced by image transmission. There was evidence of clinical utility in remote hospitals and low-resource settings, although reliability was infrequently reported. Further exploratory research is required to determine minimum requirements for image quality, bandwidth, frame rate and to assess diagnostic accuracy. Clinical trials in remote settings are justifiable. Telecommunication options will depend on local requirements; no one system conveys universal advantages. The methodological quality of research in this field must improve: studies should be designed to minimise bias, and must include details of their methods to allow replication. Analysis of cost effectiveness and sustainability should be provided.

## Introduction

Medical emergencies in remote settings present specific challenges in accessing appropriate resources. Inappropriate triage, delayed diagnosis and delayed intervention can lead to patients suffering potentially avoidable adverse outcomes. Telemedicine and more specifically, telesonography has been proposed as one solution. Telesonography, alternatively referred to as *remotely supported ultrasound*, *tele-ultrasound* or *telementored ultrasound*, combines the use of ultrasound with telemedicine, which allows for off-site expert interpretation. This potentially overcomes the major disadvantage of ultrasound, namely the skill required to interpret images [1].

The process of telesonography may be conducted in two ways. It may involve synchronous transmission in which non-expert sonographer and image reviewer are linked by a real-time connection. Alternatively, it may involve asynchronous transmission where images are acquired by an ultrasound operator and are later transmitted to an expert for review [2]. Most telesonography research has focused on applications for routine examinations, particularly in obstetrics and neonatal cardiology [2]. However, there is evidence that telesonography may also be beneficial within the emergency setting, especially where there is limited access to advanced imaging. Ultrasound is inexpensive, versatile and requires few consumables [3,4]. Furthermore, it is well established in emergency medicine [5], and can be used in difficult conditions including remote or prehospital settings [6]. Meanwhile the ‘tele’ aspect of telesonography builds upon growing interest in the application of telemedicine in emergency care, which could widen access to expert advice in complex conditions such as major trauma and stroke [7–9]. However, there remains considerable controversy over the wider adoption of telemedicine technologies. This is due to concerns over quality assurance and legal regulation [10–12], as well as weaknesses in the methodology of research and a lack of evidence of cost effectiveness [7]. Therefore, we aimed to assess whether telesonography is feasible, diagnostically accurate and clinically useful in the assessment of the acutely unwell patient within the emergency setting.

## Methodology

This systematic review adhered to the Preferred Reporting Items for Systematic Reviews and Meta-Analysis (PRISMA) guidelines see S1 Table [13]. A protocol for this review was registered with PROSPERO (record CRD42016053458). Full inclusion exclusion criteria can be seen in Table 1. The principle inclusion criteria were articles that involved acutely ill or simulated patients in an emergency setting where the scan was for the purposes for detecting an emergent pathology. Included articles were published between 1946 to February 2017 in any of English, French, German, Norwegian, Danish, Russian and Mandarin. Studies which considered echocardiography alone, or used telesonography only for the detection of chronic disease were excluded, as were studies in only neonates or infants on the grounds that these modalities are less likely to be relevant to the work of rural emergency physicians. Case studies, conference proceedings, abstracts, posters and unpublished trials were also excluded. The literature search was conducted using the electronic databases: Medline, EMBASE, Cochrane Library, and CINAHL from 1946 up to the present day. A specific search strategy for each database was constructed using MESH and free text terms. The complete form of the search strategy for Medline is listed in S2 Table. Searches of Google Scholar and Scopus were also conducted, as well as a review of included studies’ citations and references.

**Table 1:** Inclusion/Exclusion criteria.

Domain	Inclusion	Exclusion
**Study type**:	Any of: prospective observational studies, case series, feasibility study, clinical setting, economic analysis, randomised controlled trial. Plus: published in a peer reviewed journal and full text.	Any of: case studies, conference proceeding, abstracts, posters, full text not available after request, unpublished trials.
**Participants**:	Any of: human, clinical setting, acutely unwell patients, simulated patients. Plus: operators separate from image assessors.	Any of: Neonates, infants.
**Setting**:	Any of: Emergency medical department, prehospital setting, hospital wards, remote and rural clinic likely to be the sole access to medical care in the case of an emergency.	Urban general practice.
**Procedure**:	Any form of ultrasound scan. Plus: sonographer and expert image reviewer in separate locations. Plus: sonographer or reviewer involved in the provision of emergency medical services. Plus: scan for an indication directly relevant to the emergency services Plus: scan for the purposes for detecting an urgent and serious pathology/where it is probable that the findings of scans would require same day change in clinical management.	Any of: Scans acquired and interpreted by the same practitioner. Sonographer and reviewer unlikely to be involved in the provision of emergency medical services. Scans performed for indications that would not be performed in an emergency medical situation. Scans in which it is highly unlikely that the outcome would require a same day change in clinical management. Studies exclusively examining the use of echocardiography.
**Aims and Outcomes**:	Ultrasound for use in an acute setting with preliminary management or patient transport as the anticipated outcome of ultrasound. Plus: aims relate to the provision of emergency medical care, studies in which emergency cases were present but do not form the bulk of the patient population as long as the study outcomes and methods could be applied to an emergency setting. Plus: outcomes related to clinical utility of images or image quality.	Any of: No outcomes related to either the clinical usefulness of images, diagnostic outcome of images. Aims that are not relevant to telesonography in emergency medicine. Studies in which fewer than two patients required immediate or urgent care
**Other**:	1946 to February 2017. Plus: English, French, German, Norwegian, Danish, Russian, Mandarin.	Pre 1946. Other languages

### Selection of studies, data extraction and management

The citations acquired from the search were managed through Refworks (ProQuest LLC, USA). Titles and abstracts were screened by a single researcher, GMF. Following the selection of appropriate abstracts, full texts were independently screened by two researchers against the inclusion/exclusion criteria (GMF and LE). The researchers discussed their decisions to ensure consensus was reached. A third independent reviewer (PW) adjudicated and resolved disputes. Following the identification of the relevant full text articles information arising from the eligible studies was collated (see data extraction form S3 Table).

### Assessment of risk of bias

The methodological quality of the studies was assessed using an amended form of the QUADAS-2 checklist (revised tool for the Quality Assessment of Diagnostic Accuracy Studies, S1 File) [14]. Seven domains were considered in assessing bias: 1) Generic quality standards; 2) Participant selection bias; 3) Bias in the conduct of the index test; 4) Bias in the conduct of the reference standard; 5) Flow and timing; 6) Telemedicine specific reporting items; 7) Applicability of the evidence. Selected studies were independently reviewed by two researchers and disagreements were discussed and resolved through consensus.

### Data synthesis

It was not possible to conduct a meta-analysis using this data, due to high risk of bias in included studies and significant heterogeneity in study designs and outcome measures. Instead, descriptive summaries were generated. Confidence in the accumulated body of evidence was assessed qualitatively but was not graded.

## Results

**Fig 1:**
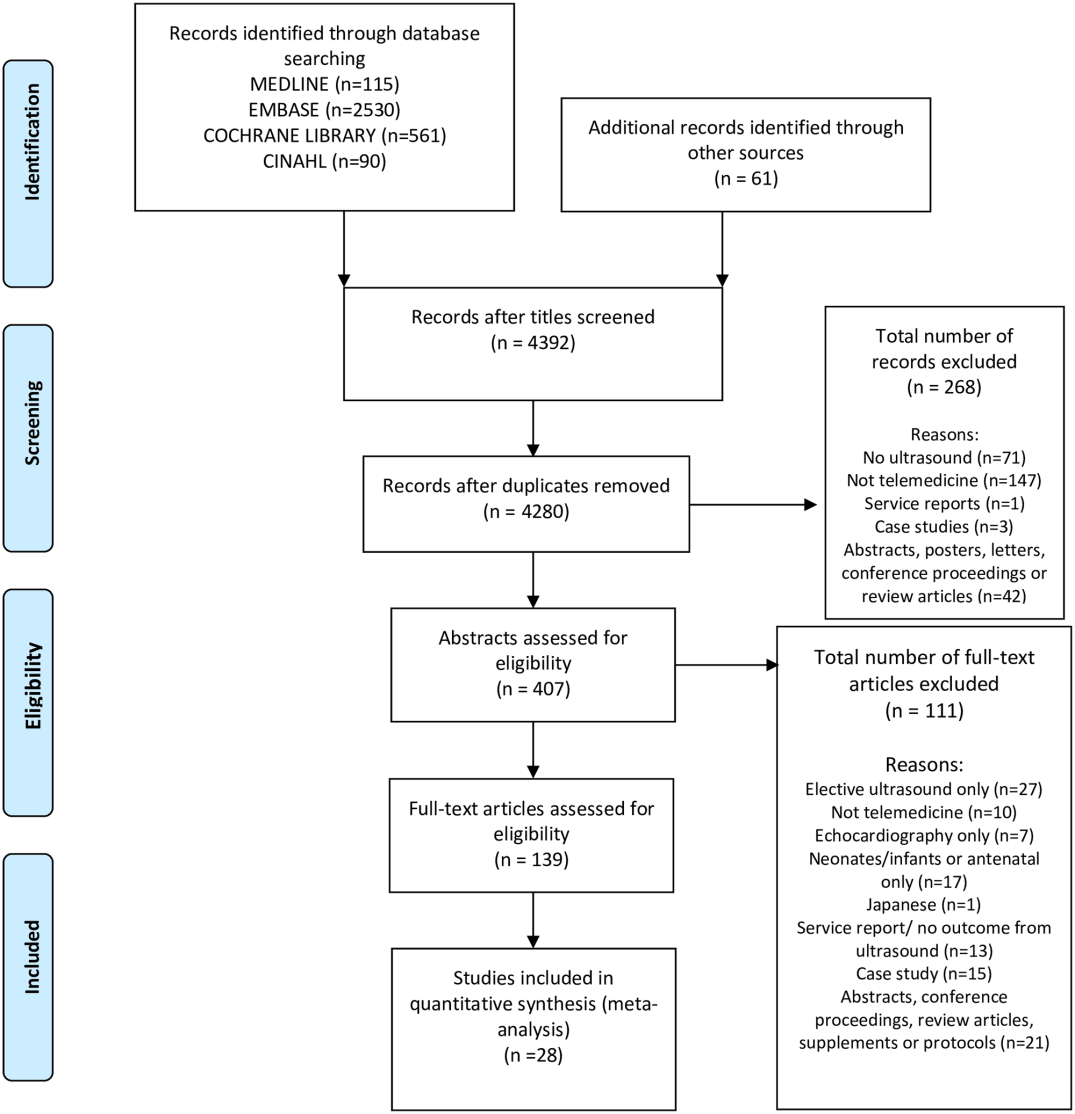
PRISMA flow diagram. Flow diagram demonstrating the selection of studies included in this review [13].

Database searching obtained 4331 references, 61 additional articles were found following reference and citation screening. After screening against the inclusion criteria 28 papers were included [15–42]. The PRISMA flowchart diagram can be viewed in Fig 1.

### Study design and methodology

The included papers were highly heterogeneous in design, quality and outcome, see Table 2. The majority of papers could be divided into three types. Firstly, feasibility studies considering the capability of a range of technologies to transmit and receive clinically useful ultrasound images [16,18, 20, 21, 23, 28– 41]. Secondly, diagnostic accuracy studies that considered the diagnostic capabilities of telesonography [19, 25, 26, 42]. Thirdly, studies considering the experimental application of telesonography in a clinical context without the use of randomisation or control groups, which we will refer to as *pilot studies* [15, 22, 24, 27]. Study size was variable ranging from 1 to 200 patients. Studies which included only one patient nevertheless involved multiple operators or remote experts hence were classed as feasibility studies. It is worth noting that the papers by Al-Kadi et al. [17], and Dyer et al. [22] concern the same telesonography system. A more detailed description of study methodology can be found in S4 Table.

**Table 2:** Study characteristics.

ID	Study design	Summary of authors’ conclusions
Adambounou 2014	Pilot study	Satisfactory image results were obtained using a low cost telesonography platform in a low-resource setting.
Adhikari 2014	Feasibility study/simulation	In a simulated disaster triage setting, it was possible to transmit, receive and interpret ultrasound videos of FAST examinations using a pair of mobile phones.
Al-Kadi 2009	User survey	Following a trial of telesonography during resuscitations in a remote hospital, the majority of staff considered telesonography to be a useful clinical and teaching tool, and could provide benefit for trauma patients.
Biegler 2013	Feasibility study	Nurses can effectively detect post-chest tube removal pneumothoraces using telesonography. The essential technical elements are Internet connectivity and a remote expert.
Blaivas 2009	Diagnostic accuracy study	The image quality, detail and resolution of ultrasound pictures recorded by one phone and then sent to another were not significantly different to high-resolution thermal printouts. However, measurements were too small to be read and reviewers had significantly lower confidence in making a diagnosis when using ultrasound images viewed on a phone.
Boniface 2011	Feasibility study/simulation	When given brief teaching on ultrasound use paramedics with no previous experience of ultrasound could successfully perform a tele-mentored bedside FAST examination.
Courreges 2005	Feasibility study	A robotic telesonography was designed and tested by two medical teams on 52 patients. Diagnoses using robotic telesonography were in concordance with results from conventional scans in 80% of cases. Conditions in which legions were small or subtle caused more difficulty
Dyer 2008	Pilot study	A telesonography system for use in major trauma cases in a remote hospital in Canada was found to be technically and clinically feasible, and enabled confirmation of diagnoses of haemoperitoneum and pneumothorax.
Ito 2013	Feasibility study/simulation	A wearable, portable telesonography robot was designed to perform FAST scans in emergency situations. This robot telesonography system was capable of capturing and transmitting ultrasound scans even during body motions such as rough breathing and coughing fits.
Johnson 1998	Pilot study	A telesonography service between remote location and a large hospital in Alberta was found to be feasible. Furthermore, remote supervision was found to be as effective at generating a reliable diagnosis as on site supervision, and the use of telesonography avoided transfer in 42% of cases and influenced management in 59% of cases.
Kim 2015	Diagnostic accuracy study	Telementored telesonography could effectively diagnose paediatric appendicitis, with a comparable degree of accuracy to in person expert ultrasound and a higher degree of accuracy than ultrasound performed by residents without mentoring.
Kim 2016	Diagnostic accuracy/Feasibility study	A smartphone can be used to accurately diagnose of the presence of paediatric appendicitis as well as to remotely interpret left ventricular ejection fraction.
Kolbe 2015	Pilot study	Didactic teaching, practical application and telementoring, can be used to support and disseminate POCUS in remote and rural areas worldwide.
Kwon 2016	Feasibility study/simulation	The provision of minimal training, and support of remote telementors allowed novices acquired diagnostic quality musculoskeletal ultrasound assessments.
Lee 2016	Feasibility/diagnostic accuracy study	Telementoring can allow novice ultrasound operators to perform scans in order to make difficult diagnoses, such as appendicitis, as effectively as onsite mentoring from an expert.
Levine 2015	Feasibility study/simulation	Using a tele-ICU system non-physicians with minimal training, can be telementored to obtain diagnostic quality images. These images can be sent without image degradation, so for most anatomic sites transmitted images are of equivalent clinically utility to those directly from the ultrasound device.
Levine 2016	Feasibility study/simulation	Low cost software can be used to transmit ultrasound images that are of high quality and that are clinically useful. Images transmitted using these methods are equivalent to images directly obtained from the ultrasound device in almost every anatomic location assessed.
Litelpo 2010	Feasibility study/simulation	The use of low cost software is able to facilitate transmission of real-time ultrasound video to an iPhone. Images can be transferred without loss of image quality and with minimal delay. Smaller delays were experienced when Wi-Fi rather than 3G connections were used.
Litelpo 2011	Feasibility study/simulation	The transmission of ultrasound video clips using wireless connections, inexpensive hardware, free videoconferencing software and domestic internet networks is feasible. It is possible to retain a standard of image quality sufficient for interpretation. Wi-Fi transmission results in less image degradation than transmission by a 3G network.
Macedonia 1998	Pilot /feasibility study	The quality of the images recovered from transmitted 3D ultrasound datasets was dependent of factors such as patient morphology and recent meals, but independent of the operator’s level of training. The authors felt that the technique had limited value without the addition of features including Doppler and real time volume acquisition.
McBeth 2011	Case series	It is possible to conduct telesonography using basic, low-cost commercial cellular networks.
McBeth 2013	Feasibility study/simulation	The telesonography system described allowed a remote expert to obtain and interpret ultrasound images so long as an internet connection is available.
Mikulik 2005	Pilot /feasibility study	It is feasible for novice users to perform carotid and transcranial ultrasound examinations when telementored by a remote expert.
Nikolic 2006	Feasibility study/simulation	It is feasible to train marine officers to produce diagnostically quality ultrasound images aboard ships.
Sibert 2008	Feasibility study/simulation	The use of an ambulance based telemedicine system may support rural EMS personnel and patients, although the system may be better suited for intubation than for ultrasound use.
Song 2013	Feasibility study/simulation	A telesonography system can be used to facilitate diagnosis of haemoperitoneum using a FAST scan performed by an emergency medicine technician, and this technique is about 88% accurate.
Strode 2003	Feasibility study/simulation	It is feasible to wirelessly transmit FAST from a field hospital and a moving ambulance to remote experts.
Zennaro 2016	Diagnostic accuracy study	Telementored paediatricians may perform POCUS in the emergency department guided in order to generate swift and reliable and diagnoses.

3G, Third Generation cellular network; 3D, Three dimensional; POCUS, Point of Care Ultrasound; EMS, Emergency services; FAST, Focused Assessment with Sonography for Trauma; ICU, Intensive Care Unit; tele-ICU, tele Intensive care.

Both asynchronous [15, 19, 24, 27, 34, 38, 41] and synchronous transmission with real time interaction between the operator and image interpreter [15–18, 20– 22, 24– 31, 34–37, 39, 40, 42] were reported. Several articles in a clinical setting used a combination of these approaches based on expert and operator availability [15, 34], or in cases where asynchronous scans were deemed of insufficient quality therefore a second tele-mentored scan was conducted [24]. Included studies were published between 1998 and 2016, consequently there is great variation in the system components described. The bandwidth available ranged from 0.132Kbps [19] to 59Mbps [25]. Several papers compared multiple telecommunications systems [26, 31, 33, 41]. Despite the importance of compression algorithms (which facilitate the packaging and reduction of data volume from original the file format) to successful telesonography these were not described in in the majority of studies [16, 17, 19, 20, 22, 26, 30, 34, 37, 39, 42]

### Risk of bias

**Fig 2:**
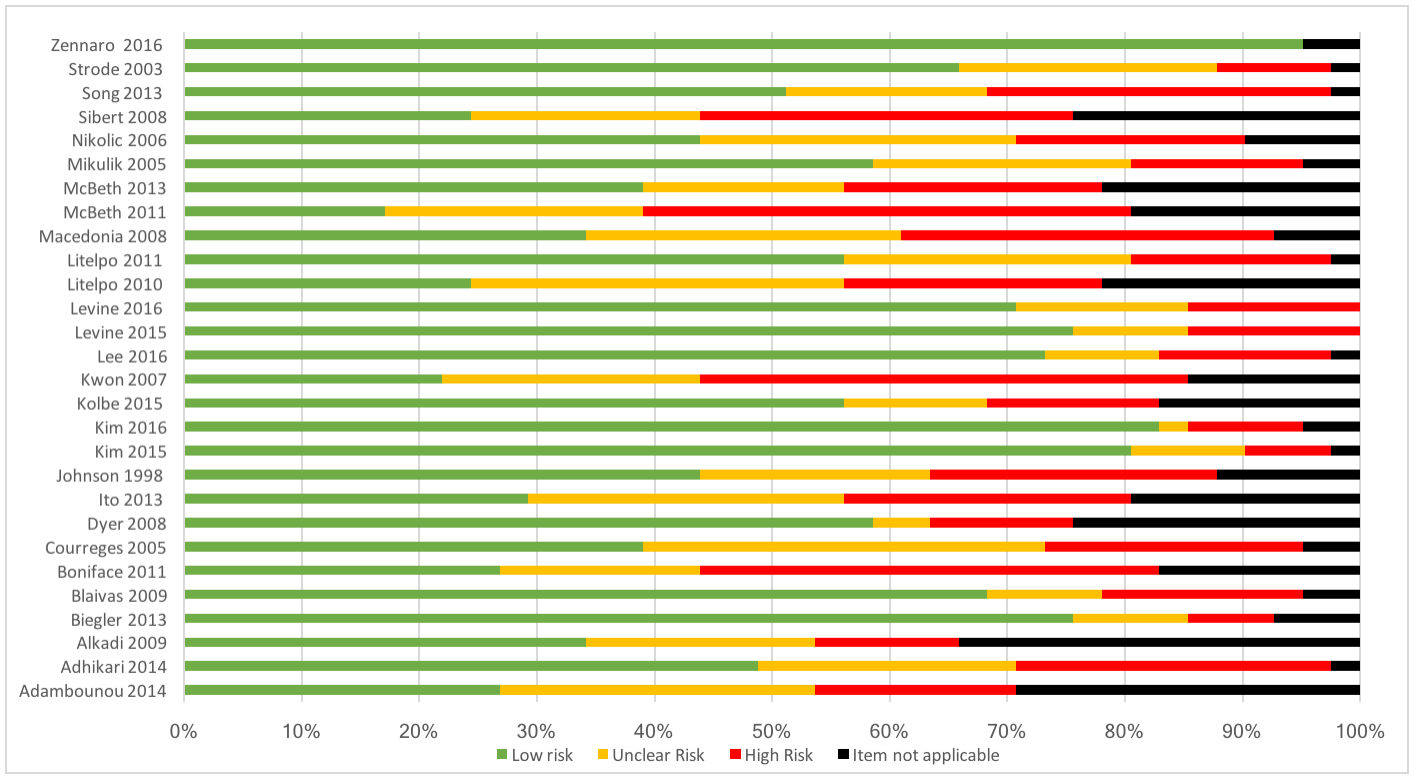
Critical appraisal results. Articles were marked as high, unclear, low risk or item not applicable according to 41 items, in the 7 domains described previously. Please see S5 Table for a domain specific representation of the risk of bias for each study.

The detailed results of the critical appraisal for each article are described in S4 Table and a summary graph can be seen in Fig 2. The overall quality of the articles was generally poor, with high risk of bias. Reporting of the technical aspects of the methodology was generally insufficient to allow replication and studies frequently lacked a reference group or comparison. Data security measures were reported in a minority of papers. Discussion was limited to a brief statement confirming the use of an encrypted communication channel and authentication systems [15, 18, 19, 25, 26, 32, 33, 42]. Phantoms, healthy volunteers and patients with chronic illnesses were frequently used as ultrasound subjects and in many instances these subjects had either gross ultrasound findings or none at all. Several studies specifically excluded overweight patients and others failed to describe patient characteristics.

The clinical applicability of the evidence was uncertain, firstly as many articles were simulations and thus were unlikely to provide an accurate representation of clinical practice. There were also concerns regarding the clinical pilot studies as only one system [17, 22] (Al-Kadi et al. and Dyer et al.) was used purely within the context of clinical emergencies, in the remainder the patient mix was likely to contain a number of patients with subacute or chronic complaints.

### Study findings

#### Feasibility

A defining feature of emergency medicine is the need to minimise delays. The time taken for scanning ranged from 4 minutes for a scan which was tele-mentored [29] to 24 minutes for a scan using a robotic device [21]; most examinations took less than 10 minutes [20, 23, 29, 30, 42]. Transmission time or other related delays were infrequently reported. No synchronous system reported a transmission delay of more than 2s [15, 22, 23, 42], and such a delay was perceived as near to real time by users [15, 22]. Amongst the asynchronous systems, lengthy transmission delays were reported ranging from 5 to 228 minutes [19, 34]. Transmission reliability was inconsistently reported, the percentage of transmissions which failed ranged through 2% [42], 14% [41], and 22% [21], and complete transmission failure was reported in one 3G-based telesonography system [33]. Inter- and intra-user reliability was considered in few studies. Nonetheless, amongst users reviewing images transmitted using telesonography agreement was moderate to high; Cohen’s kappa (k) = 0.67 [19], k = 0.80 and k = 0.82 [26] and k = 0.93 [42]. Intra-user agreement between telesonography and conventional scans was not reported.

Asynchronous scans were used in nine articles and it was demonstrated that it is possible to successfully transmit ultrasound image files from a range of locations [15, 19, 24, 27, 33, 34, 38, 40, 41]. Johnson et al. were able to demonstrate that almost all diagnoses could be made reliably from asynchronous transmission and real time video conferencing was required in only 13% of cases where uncertainty existed [24]. There is some evidence to suggest that minimally trained operators are capable of acquiring simple ultrasound scans without real time expert support [38].

Image quality was described using several different metrics including Likert scales, proportion of images of “good quality” [42], and qualitative description [24, 27, 28, 34, 35]. Of those articles which qualitatively described image quality all reported high quality, clinically useful images [24, 27, 28, 34, 35]. Of the articles which reported subjective image quality four articles reported that image quality was less than 75% of the quality of the reference standard using any given transmission technique [16, 33, 36, 39, 41]. Five articles described image quality as very good (between 75% and 87% of the reference standard) [16, 19, 21, 25, 26] and three articles as being excellent (above 90% of the reference standard) [22, 26, 42]. Several articles separately reported on the clinical utility of the images using a range of measures from the proportion of anatomic features visualised to Likert scales of diagnostic confidence [16, 22, 30, 31, 34, 36]. The judgements made concerning the clinical utility of images were generally positive with high proportions of images judged as sufficient for interpretation or identification of anatomic features [19, 22, 29, 31, 35, 36].

Many articles reported substantial issues with both image quality and transmission failure when using very low bandwidth (0.13-200Kbps) [16, 19, 21, 32–35, 39, 40]. It might therefore be suggested that to construct an effective telesonography system the absolute minimum bandwidth requirements are above 500 kbps for general use [15]. While 1–5 Mbps would provide higher quality (up 40kb per frame at 5Mbps) and enable high frame rate video review of up to 15 frames per second (fps) at 5Mbps, this may be essential for telementoring [15, 22, 25, 26]. With access to at least 24Mbps of bandwidth it could be possible to transmit high quality video, at a resolution around 150kb per image with a frame rate of 20fps [25]. In a resource constrained environment where the potential benefit of image transmission might outweigh the risk of image misinterpretation, a bandwidth of 140 kbps may be sufficient [32]. In addition, some anatomical views may not require high frame rates, for example a frame rate as low as 1.1 fps has been proposed as acceptable for the assessment of pleural sliding [32].

A cost-benefit evaluation of telesonography is essential before clinical implementation, however despite several articles alluding to ‘low cost systems’ [15, 18, 32, 35, 36, 42], only four articles explicitly listed the costs incurred [15, 19, 31, 38]. The cheapest systems used off the shelf components including phones, free software and laptops, while the most expensive systems included a dedicated tele-ICU system costing $50,000-$100,000 per bed [31]. In one study Levine et al. demonstrated that Facetime (Apple Inc., USA) was comparable to the tele-ICU system when using a good internet connection [31]. In the context of low resource settings, there was some evidence that telesonography could be implemented affordably [15]. A summary of the feasibility and reliability of telesonography can be seen in S6 Table.

#### Diagnostic accuracy

A measure of diagnostic accuracy was reported in nine studies and did not show any clear correlation with increasing image quality scores [18, 19, 21, 25, 26, 37, 40–42]. Most used conventional ultrasound scans as the reference standard. The lowest value of agreement with reference standard was recorded by Blaivas et al. who reported a moderate kappa value of 0.63 [19], Biegler et al. reported a diagnostic accuracy (effectiveness) of 92% but a sensitivity of only 66% [18] and Courreges et al. reported an 83% accuracy for diagnosing symptomatic pathology [21]. Strode et al. using four different communication modalities (two satellite systems, local area network, a vest mounted microwave transmitter) reported mixed values of sensitivity and specificity ranging between 70–82% specific and 82–95% sensitive [41]. All other studies reported a high level of accuracy: 88–100% sensitive and 87–98% specific [25, 26, 40–42].

#### Clinical utility

The utility of telesonography was demonstrated in several studies when applied within a clinical context [15, 17, 22, 24, 27]. In addition to benefits that resulted directly from access to ultrasound imaging some authors also described incidental benefits from a telemedicine link to specialists, such as advice on patient management [22]. Kolbe et al. demonstrated that telesonography was used to aid Nicaraguan staff to make new clinical diagnosis in 52.3% of patients and led to a change in management in 48% of patients [27]. Of the included pilot studies, two could be considered to be within a developing or low-resource setting and both described clinical benefit and feasibility using ‘low cost’ systems, despite some minor technical difficulties [15, 27].

Several articles also considered the qualitative reactions of medical staff to telesonography [17, 22, 30, 36, 39]. These were mostly positive, with the majority of users remarking on benefits to patients [17, 22], positive learning experience [17, 22, 30], ease of use and applicability [36]. However, the study by Sibert et al. reported that 50% of the respondents felt that the system was not clinically useful, due to image degradation, interrupted transmission and low frame rate [39].

#### Educational utility

Telesonography was considered as a teaching tool in several studies [17, 18, 20, 22, 27, 29, 30, 38]. There is evidence that it is feasible to tele-mentor or remotely support novices with medical and non-medical backgrounds [15, 17, 18, 20, 22, 27, 28, 29, 30, 31, 35, 36, 37]. Evaluative work comparing tele-mentoring to onsite mentoring suggests that there is no significant difference between time taken and success rate of image acquisition between onsite and remote mentoring [29]. Two papers found that telesonography with remote mentors had almost equivalent sensitivity and specificity to conventional ultrasonography in a range of scenarios [25, 42]. The use of tele-mentored telesonography was described as beneficial in clinical practice in several instances [15, 17, 22]. Furthermore, the use of tele-mentoring was viewed in a positive light by both physicians performing and observing telesonography [17, 22] and improved the skills of participants [17, 22, 27, 30, 36, 39]. The use of minimally trained operators and asynchronous transmission resulted in a low proportion of usable images: 73.3% [38]. In all articles in which didactic teaching or telementoring was provided there was minimal description of the protocols and supportive materials used and there was concern over the methodological quality of a subset of articles assessing telementoring.

### Evidence according to system characteristics

#### Patients, pathology and setting

Most papers in this review have included a proportion of patients found outwith the emergency setting. As a consequence studies may contain findings that cannot be replicated when the additional challenges of pure emergency medicine are imposed. Nonetheless, there was evidence from these articles that telesonography could be used to identify variety of abnormalities relevant to emergency medicine such as the presence of peritoneal fluid or pneumothorax [15, 18, 21, 24, 27, 29, 30, 31, 34, 38]. Only one telesonography system was used in the setting of resuscitation and in this case it was reported that telesonography was a beneficial addition to the emergency department [17, 22]. Paediatric cases were considered in two articles [25, 26, 42] which found strong evidence to support the feasibility of tele-mentored ultrasound, although this was somewhat less sensitive (88.2% sensitive as in-person expert scans), and specific (99.7%) than conventional methods [42]. A range of scan views were considered and the evidence within each of these is discussed in Table 3.

**Table 3:** Summary of evidence according to system characteristics.

**Mode**		
**Tele-mentoring+ Synchronous**	**Synchronous**	**Asynchronous**
**Requires**: Feasibility studies determining minimum bandwidth requirements and definitive RCT. **Evidence**: This mode may need a high bandwidth, most effective studies had access to > 1Mbps [22, 25, 29, 42]. However, more work is needed to clarify this in order to allow researchers to assess the suitability of this technology to their setting. Good evidence of feasibility albeit from small studies of mixed quality [15, 18, 20, 27, 28, 29, 31, 34, 35, 36, 37, 42]. There is also good evidence of diagnostic accuracy from a small number of strong studies [25, 42]. There is some evidence of clinical utility from pilot studies of mixed quality [15,17, 22, 27].	**Requires**: Pilot studies in clinical setting with comparator groups. **Evidence**: Good evidence of feasibility albeit in a number of small studies [16, 32, 33, 39, 40]. Some evidence of feasibility with low bandwidth [<200kbps] [32, 33, 39, 40]. Some mixed evidence of diagnostic accuracy [26, 40]. Clinical utility has not yet been demonstrated and it is unclear what the role of this mode of transmission without the addition of tele-mentoring.	**Requires**: Definitive RCT **Evidence**: Strong evidence feasibility of this method in a large number of small studies [15, 19, 24, 27, 34, 38, 41]. Some evidence that this is possible with very low bandwidth [<200kbps] [16, 19, 34]. Good evidence of clinical utility [15, 24].
**3D**	**Robotic**	**Remote task scale**
**Requires**: Feasibility studies, with comparison with to other transmission modes. **Evidence**: Two small pilot studies, poor quality evaluation of method but some potential of clinical utility demonstrated [15, 34]. Wider literature suggests potential in this field.	**Requires**: Theoretical and feasibility work to establish superiority. **Evidence**: Poor quality feasibility studies [21, 23] have not established yet that robotic devices convey advantages when compared to tele-mentored telesonography in an emergency setting.	**Requires**: Feasibility studies. **Evidence**: No evidence found in this review, some evidence in the wider literature.
**Setting**		
**Rural emergency department**:	**Low-resource setting**:	**Prehospital**
**Requires**: Definitive RCT. **Evidence**: Evidence in the wider literature of clinical need for such a technology. Good evidence of feasibility [17, 22, 42] and diagnostic accuracy [26, 29, 42]. A flexible approach utilising asynchronous and synchronous transmission modes dependent on local requirements and physician availability may be most appropriate.	**Requires**: Pilot studies and Definitive RCT. **Evidence**: Evidence in the wider literature of clinical need, however more assessment of the appropriateness of telesonography to the local setting is required before conducting research. Little evidence of ability to quality control and obtain usable images [15, 27]. Some evidence of clinical utility and accuracy using telementored scans [15, 27]. Also must be considered in the context of other urgent and severe healthcare needs.	**Requires**: Feasibility studies and pilot studies. **Evidence**: More work required to develop optimal transmission system, evidence of problems with reliability of transmission [17, 26, 27, 29, 30, 33, 35] particularly from moving vehicles [33, 34] clinical benefits of prehospital ultrasound have not yet been established by the wider literature.
**Tele- communications available**		
**VPN/WAN/LAN**	**Cellular/Wireless connection**	**Satellite connections**
**Requires**: Incorporation into RCT. **Evidence**: Feasible to use telesonography using fixed line internet connection [42]. Feasible provided sufficient bandwidth. Shown to be clinically useful using custom VC software [17, 21, 22, 34, 37, 39, 42]. Possible using voice over phone alone [20, 28, 29]. Security may be greater with this mode of telecommunications. May be superior option is synchronous connection is required.	**Requires**: Incorporation into pilot studies. **Evidence**: Feasible using cellular [19]. Feasible using cellular connection, mixed image quality [32, 33, 39, 40]. Good evidence feasible with strong Wifi connection [15, 18, 31]. Feasible using free VOIP software [15,18, 27, 31, 35, 36].	**Requires**: Incorporation into pilot studies **Evidence**: Using a satellite connection, mixed image quality [19]. Mixed results regarding Image quality. May require substantial time for transmission [34].
**Specialty/Indication**		
**Paediatrics**:	**Neurological**:	**Trauma**:
**Requires**: Definitive RCT. **Evidence**: Good quality of evidence of clinical utility, and diagnostic accuracy [25, 42].	**Requires**: Feasibility studies and Diagnostic accuracy studies. **Evidence**: Potential utility in a number of settings, some evidence feasibility and diagnostic accuracy, however the strength of evidence is currently insufficient [37, 39].	**Requires**: Definitive RCT. **Evidence**: Using synchronous transmission and tele-mentoring there is evidence of feasibility [20, 22, 32, 33, 35, 36] some evidence of clinical utility [17, 22]. Using asynchronous transmission alone there is a lack of evidence, perhaps worthwhile exploring if the transmission time is brief and there is sufficient supporting information. 3D techniques may decrease risk of inexperience resulting in inadequate visualisation.
**Lung**:	**Musculoskeletal**:	**Appendix**:
**Requires**: Diagnostic accuracy and pilot studies. **Evidence**: Some evidence of feasibility [17, 18, 22, 35, 36], and some evidence of clinical utility [17, 22]. Using Synchronous alone methods alone it may be feasible to transmit lung sliding data, but may require higher frame rate and therefore a higher bandwidth connection [32, 33].	**Requires**: Pilot studies in clinical setting. **Evidence**: Evidence of feasibility but not clinical utility [28].	**Requires**: Definitive RCT. **Evidence**: Strong evidence for the efficacy of telementored ultrasound with high bandwidth and good quality [25, 29].

RCT, Randomised control trial; VOIP, Voice over internet protocol; VPN, Virtual private network; WAN, Wide area network; LAN, Local area network.

There were also a number of simulation studies which considered less conventional field hospital and prehospital applications of telesonography [16, 23, 28, 34, 35, 36, 38, 39, 40, 41]. Of these several included studies of telesonography during patient transport in ambulances [39, 40, 41] and in flight [35]. Outcomes including image quality and transmission reliability suggest it may be problematic to transmit real time images from an ambulance [39, 40, 41]. In general, these studies were of poor methodological quality and were limited by small sample size and the use of simulated patients.

#### Transmission technology

Studies using satellite transmission had mixed results [21, 34, 41]. These papers predominantly used asynchronous transmission and incurred significant transmission delays [34, 41] although this varied according to the satellite system used [34]. There is limited evidence of reliability as variable levels of image degradation were reported and this resulted in reduced sensitivity and specificity [4].

There were a number of articles in which cellular transmission was utilised for all or part of the data transmission route [16, 19, 21, 25, 26, 29, 32, 33, 35, 36, 39, 40]. In articles which used 3G networks there were mixed outcomes and several authors had difficulties successfully transmitting useable images [32, 33, 39]. However, other studies did manage to transmit high quality images [26, 35, 36] and were most successful when high bandwidth (55-59MBps) cellular connections were used [25, 29]. It was noted that transmission reliability may be improved by the use of a communications management system to withstand bandwidth fluctuation [40]. A number of articles used Wi-Fi connections [15, 18, 30, 31, 32, 33, 42]. Where this was compared with 3G cellular connections, Wi-Fi produced reduced image degradation and transmission loss [32, 33]. However, several articles purely investigated transmission using a local, hospital Wi-Fi connection, which was likely to be more stable than over a larger area [18, 30, 31, 42].

#### Ultrasound variants

3D ultrasound image acquisition equipment was used successfully to obtain and transmit images in two studies [15, 34] although significant time was required to transmit the images due to the large volume of data [34].

Robotic ultrasound remains a theoretical possibility but has not yet been proven to be effective [21, 23]. Courreges et al. reported a diagnostic accuracy of 80% for symptomatic pathology on ultrasound recorded by their robot, however there were also multiple instances of scan failure and difficulties surrounding the visualisation of small legions [21]. Furthermore, Ito et al. described a lengthy attachment procedure to properly fit the robot to the patient [23].

#### System components

Several studies investigated the use of images recorded by a secondary device, such as a mobile phone or video camera pointed at the screen of the ultrasound machine [16, 19, 30, 31]. In these articles a surprisingly high subjective image quality was seen, ranging from 7.5 to 7.9/10 [16, 19]. The remainder of the articles used systems in which the original image data from the ultrasound machine was transmitted either through ultrasound machines with inbuilt communication technologies or through intermediate devices [15, 18, 20, 21, 22, 23, 24, 25, 26, 27, 28, 29, 32, 33, 34, 35, 36, 38, 39, 40, 41, 42]. The hardware used for receiving and viewing scans remotely included mobile phones [16, 19, 26, 32], and portable laptops [15, 18, 30, 31, 33, 35, 36, 41, 42). There was a range of ultrasound models used and the majority of studies employed portable devices [16, 18, 20, 27, 28, 30, 31, 34, 35, 36, 37, 38, 39, 40, 41]. There was no obvious disadvantage to the use of these portable devices, although some models lacked spectral Doppler or M-mode capabilities [34, 35, 36]. It was demonstrated that free Voice over Internet Protocol (VOIP) software can be used [15, 18, 27, 32, 33, 35, 36] although a custom videoconferencing (VC) system may provide a more stable connection and stronger security options [15, 17, 21, 22, 23, 24, 37, 39, 42].

## Discussion

### Summary of main results

Despite heterogeneous literature with substantial risk of bias, telesonography is feasible in a wide range of settings and for a wide range of indications. Furthermore, the diagnostic accuracy of telesonography has been shown to be comparable to conventional in-person ultrasound in emergency situations where there are overt ultrasound signs. Additionally, novice operators may, in some situations, be successfully tele-mentored to produce clinically usable ultrasound images in the acute setting. Both real time and delayed (asynchronous) ultrasound diagnosis can be useful in the clinical setting, however the impact of telesonography on patient outcomes has not been assessed. There was no single telesonography system that conveyed superior benefit in all circumstances, and no particular disadvantage seen when smaller screens or portable ultrasound machines were used. In remote locations telesonography may be a useful addition to a portable clinical toolkit and may provide benefit in emergency and elective cases. However, there is insufficient research on the applications in resuscitation situations and in prehospital care. Included studies were limited in size and scope and future research on this topic should consider a more rigorous approach to the design of feasibility studies, as well as the reporting of technical aspects and study limitations in order to build a more cohesive and applicable body of literature.

#### Applicability of these findings

The principal finding of this review was that telesonography is feasible in a range of circumstances. How widely this conclusion might be applied is more contentious. Almost half of the world’s population lives in remote regions, which are disproportionately affected by poor telecommunications coverage [43]. Nonetheless, even in the least developed countries the majority of internet connections are capable of between 256 kbps and 2 Mbps therefore telesonography is theoretically possible [44, 45].

Of the telesonography systems available, asynchronous transmission may be the most widely accessible form. This way images of diagnostic quality can be sent over less reliable communication channels with a lower bandwidth, as transmission delays and interruptions are less relevant. This mode might also pose fewer financial and logistic difficulties (especially in terms of scheduling time with remote experts). Furthermore, Johnson et al. found that the number of cases in which an additional synchronous scan was needed to clarify the findings of an asynchronous transmission was ~12% and this provided a new diagnosis in only 1% [25]. If these results are replicated and synchronous transmission is shown to provide limited additional benefit asynchronous transmission may often become the preferred mode of telesonography, particularly if there is capacity for the occasional use of synchronous transmissions in cases of uncertainty.

However, some investigators remain sceptical of the asynchronous approach and in a review in 2010, Meuwly concluded that asynchronous transmission was unsuitable for emergency situations or for cases where local operators lack experience. Undoubtedly, given the dearth of available professionals in remote and under-resourced areas, recruitment of skilled operators may be the chief barrier to asynchronous telesonography [46]. Existing WHO training recommendations involve substantial time and resource commitments for ultrasound trainees (3–6 months and 300–500 ultrasound examinations for physicians) [47]. Training standards in emergency medicine are less clear, and may be less intensive if the need for interpretation was entirely removed. Given the evidence in this review and the wider literature that suggests that a wide range of individuals can execute simple scan protocols further research on the efficacy of the use of minimally trained operators is warranted.

It has been proposed that the use of 3D scans, which do not require the operator to understand the interaction between the 2D ultrasound beam and the 3D anatomy, will reduce the risk of novice operators acquiring inadequate ultrasound images [48]. A review in 2011 of 3D ultrasound for novices found that 3D ultrasound may be superior to 2D imaging techniques in improving the reliability of measurements of irregular organs, reducing both the time required for scanning and the need for rescanning of patients [48]. However, only two papers used this technique within this review and there was no critical evaluation of the relative effectiveness of this method within emergency medicine [15, 34]. Therefore, it is conceivable that it may be useful within emergency medicine, and also merits further investigation.

Another key barrier to successful application of asynchronous transmission is the potential for loss of important information regarding the clinical context. This may decrease the applicability of advice provided and has the potential to increase diagnostic errors, as has been demonstrated in a study of emergency teleradiology services in the UK [49]. This issue may partly be ameliorated by the use of synchronous transmission that allows for personal interaction between remote experts and local staff. This may simultaneously facilitate additional broader interactions regarding patient management and allow for clinical teaching [22, 27, 50]. Such a system might also provide cost savings by allowing ultrasound operators to learn ‘on the job’ as there is evidence that a short training duration may be sufficient for operators to acquire ultrasound images under remote guidance [51]. In areas where communications are insufficient to support real time transmission of full quality images, an alternative option is *Remote Task Scale*. This technique involves the real time image transmission of low quality images followed by asynchronous transmission of full quality images [2] but was not found in any papers in this review.

### Telecommunications requirements and choices

The choice of telecommunications method must be made based on the requirements of the locality and the system. Despite 20 years of telesonography research the image standards essential for diagnostic utility have not yet been defined. Some researchers have suggested bandwidths ranging from 384 kbps to 0.6 Mbps as the threshold for the maintenance of diagnostic image quality, depending on the indication and anatomical view required [2, 52]. However, emergency medicine scans may not require the same resolution as specialised scans looking for subtle anatomical defects. Therefore, it is necessary to determine threshold values for acceptable image quality across a number of scan types specifically for use in emergency medicine.

Fixed line broadband connections are likely to offer the most stability and the highest bandwidth, particularly if advanced technology such as fibreoptic cables are used. If available, the security and stability offered by LANs, WANs or virtual private networks (VPNs) may be preferable. In low-resource settings, the most universally accessible communications systems currently available are mobile/cellular networks and these may be a good option where adequate bandwidth and security measures are available. An alternative is satellite broadband and this may be the only viable solution in extremely rural areas. It is likely that as the affordability, coverage, reliability and bandwidth provided by satellites continues to improve they may become an increasingly attractive option for telemedicine [53]. This may be particularly important for emergency medical transmissions in the case of damage to terrestrial antennas, wired connections and ground-stations, as may occur during a natural disaster or humanitarian crisis [54].

### Comparison to current literature

Despite their scarcity and small size the results from the included pilot studies of telesonography between hospitals were promising, but there is a need for more robust studies to be conducted before this is routinely used in clinical practice. These findings are in line with the conclusions of a series of narrative reviews on general telesonography published between 2004 and 2015, which found no substantial evidence of benefit to patients. All found marked heterogeneity in the design of the included studies and all recognised the need for further more definitive research [2, 47, 52, 55, 56, 57].

This review found that in studies where telesonography was used in hospital emergency departments some clinical benefits were seen and a positive response from involved staff was reported. This echoes the conclusions of a systematic review of general telemedicine use in the emergency room, which found considerable benefits for small hospitals with a low frequency of emergency cases. These included improvements in staff satisfaction, clinical processes and outcomes [57]. Given the importance of clinicians’ attitudes to the integration of technology in clinical practice these opinions are important endorsements of telesonography and may help guide the design and implementation of systems. Therefore, in future studies of telesonography qualitative process analysis should be encouraged [55].

Given the competing demands placed on resources within emergency medicine, a quantification of the local requirement for diagnostic ultrasound and telemedicine is essential. This has yet to be formerly assessed, however there is some evidence for existing unmet need for ultrasound imaging in rural emergency departments [58, 59]. This may be due to lack of training, difficulty with image interpretation, and lack of support personnel, as well as skill atrophy due to infrequent use [60]. In this context, telesonography, particularly synchronous telesonography, addresses the majority of these concerns. Therefore, the use of telesonography in rural emergency departments is a logical step towards increasing diagnostic facilities in isolated areas, and progression of research into definitive randomised control trials should be considered in situations where there is a reasonable expectation of clinical benefit. Similarly, remote and rural general practice, travelling doctors and community health centres may also benefit from a telesonography [24]. Given the increasing pressures on hospital emergency services, and a drive to decrease unnecessary emergency admissions in many countries this area also warrants further research.

There is some evidence that telesonography could be beneficial in a low resource setting, a conclusion which mirrors the findings of related narrative reviews [56] and reviews of conventional ultrasound in the developing world [61]. In the absence of the capacity to support more advanced imaging techniques the addition of telesonography may have a significant clinical benefit. However, it is also in this setting the capacity for establishing telecommunications and conducting research may be most limited. Study designs which facilitate flexible access for both acute and chronic indications may be most appropriate, together with emphasis on ultrasound techniques which are relevant to local epidemiology [62]. In this setting a multipurpose telecommunications network which could provide decision making support, remote prescribing and staff training may be most appropriate and might contribute to a reduction in the professional isolation of remote practitioners [63]. However, enthusiasm for the theoretical benefits of technology must not overtake the evidence and evaluation of practical barriers [64], including consideration of long term requirements such as electricity and maintenance [62]. Furthermore, ultrasound training and use is infrequently regulated in such low resource settings, therefore the risk of harm from improper use may be greater in this context, and as such measures to ensure the consistency and quality of ultrasound use should be built into study design [61]. Given the potential benefits as well as the complexities of telesonography implementation in this setting, it would be beneficial to perform thorough needs assessments, an assessment of readiness, pilot studies and qualitative work specific to the locality before conducting costly trials.

There was some exploratory work within prehospital telesonography for the emergency services in this review. However, the benefit of telesonography is more questionable in this setting and it has yet to be established that ultrasound, even performed by an expert on site, provides substantive benefit [65]. Specifically, a recent systematic review by O’Dochartaigh and Douma suggested that the evidence was moderately supportive of prehospital ultrasound in trauma patients [66]. Problems which were highlighted as significant in the prehospital arena largely concern practical difficulties in acquiring or interpreting prehospital ultrasound images [6, 67]. As a result, theoretical benefits remain, particularly if patient transport is likely to extend over a long period or in conditions where prehospital management is possible. However, this area would benefit from further exploratory studies to identify successful telesonography system designs for ambulances, and pilot studies both to estimate the clinical benefit of such systems and quantify risk of harm due to delays in patient transport.

The applications for telesonography in stroke were not adequately explored within the articles included in this review. Rapid access to expertise is arguably more important in stroke than any other medical presentation given the short therapeutic window for administering thrombolysis. Therefore, there is substantial interest in developing telestroke services [68, 69]. However, evidence for the value of transcranial Doppler in the differentiation of ischaemic or haemorrhagic stroke is currently insufficient. It is only demonstrated to have a high sensitivity and specificity in the anterior circulation [70]. Transcranial Doppler is currently limited in its use as an adjunctive procedure to monitor the effect of thrombolysis and to assess the risk of recurrent events [71, 72]. Furthermore, 10% of patients may be unsuitable for scanning through this method due to lack of a suitable bone window to allow visualisation of anatomy [73, 74]. Nonetheless, further exploration of ultrasound for stroke is warranted. In the prehospital setting portable ultrasound tools may facilitate the identification and transfer of patients with large vessel occlusion to a stroke centre with access to endovascular recanalization therapy [75]. Furthermore, low cost tele-neurosonography may have particular relevance to low and middle income countries where the incidence of stroke is particularly high [76].

### Costs and consequences

The cost effectiveness of any new intervention must be carefully considered and there was no formal cost-benefit analysis conducted in any paper in this review [77]. Furthermore, alongside the costs of equipment maintenance the sustainability of telesonography from a human resource perspective should be considered [78]. No study in this review evaluated the impact of the additional workload on remote experts or ultrasound operators. This is a concern which is particularly relevant within the resource constrained field of emergency medicine and has the potential to cause patients harm should scanning or transmission detract from the essential elements of emergency care.

The ethical and medico-legal management of errors must also be considered carefully, as should issues of reimbursement, patient data safeguarding and confidentiality [79]. Additionally, even with the most technologically advanced transmission systems, remote interpretation has a slightly reduced sensitivity and specificity compared to in person interpretation. Given the already variable accuracy of conventional ultrasound this may result in a tool with unacceptably low sensitivity, which is particularly concerning if telesonography is misinterpreted as a definitive diagnostic tool or if telesonography fails to incorporate the transfer of contextual clinical information. Moreover, while increasing access to diagnostic imaging may be beneficial, it may also reveal incidental findings, therefore goal-directed *point of care ultrasonography* (POCUS) may be more appropriate than the exploratory approach of classic consultative ultrasound [1]. Lastly, there is a danger when considering any telemedicine-based intervention that it might be seen as a replacement for adequate resourcing of local services, and given this telemedicine should be viewed as a supportive rather than a substitutive tool [64].

It is essential that the quality of research in this field improves. To this end there are several areas which researchers should focus. Firstly, studies should aim to either replicate or build upon the existing literature (Table 3 highlights how this might be achieved). All studies should also refer to existing reviews of sonography, telemedicine and telesonography that relate to their proposal and use these to clarify their research objectives. To inform the design of a novel telesonography system a theoretical model of telesonography (as proposed by Sharon and Frank, 2000) may be helpful [80]. In selecting the most appropriate outcome measures and methodologies researchers should consider referring to existing guidelines on the evaluation of telemedicine, such as those by the Lewin group and Institute of Medicine (IOM) [81, 82]. Particular care should be taken to ensure study validity when designing feasibility and pilot studies [83]. In most cases the inclusion of a reference group or comparison can improve validity. Furthermore, as the rationale of a pilot study is to investigate areas of uncertainty to inform a future definitive RCT, this should be stated within the aims of the study and should be followed by an RCT. In all study types the selection of appropriate outcome measures is essential. An exploratory RCT or concurrent validity study could include outcomes such as: number of new diagnoses made, change in patient management attributable to telesonography, time to definitive diagnosis, time taken for scan and transmission.

Through all stages of study design and conduct every measure to minimise bias should be taken, irrespective of study size. To this end it is essential to ensure that reviewers of ultrasound images or system assessors are independent. In many studies it will be essential to have some objective judgements of the diagnostic accuracy of telesonography: a protocol for the objective evaluation of this is recommended by Hemmson et al. [84]. Investigative operating environments should replicate the environments used in practice. For example, pre-recorded scans from an emergency department may be more appropriate than those from chronically unwell patients if the study aims to assess transmitted image quality and diagnostic accuracy. The reference standard used should be standard practice, e.g. conventional scans conducted by an expert. In order to improve the standard of telesonography literature authors should refer to reporting guidelines [13]. Diagnostic accuracy studies could refer to guidelines such as Standards for Reporting of Diagnostic Accuracy (STARD) or QUADAS-2 [14, 85]. When discussing studies conducted in the clinical environment authors should consider the applicability of their system from multiple perspectives (including the patient, healthcare provider and organisation); frameworks are available to aid this process [81, 82, 86, 87]. All authors should also consider the limitations and applicability of their telesonography system. Table 4 highlights the salient reporting items required for telesonography research, with the aim of encouraging methodological standardisation and validity in this setting.

**Table 4:** Suggested reporting items in telesonography studies.

Patient and Participants	Technology	Results	Discussion
**Patient/Subjects**: Number (n), Power calculation. **Other participants**: training and experience of ultrasound operator/reviewer, blinding of reviewers, participant role/interest in telesonography outwith study. **Baseline characteristics**: Age, BMI category, Pathology. **Specific features**: Severity of anatomic defect, Injury severity scale for trauma. **Procedure**: Sequence of events involved in transmission. **Medico legal**: Encryption and data protection procedures used.	**Scan type**: Full scan protocol. **Equipment/components**: Make, model type, probe types, software and hardware components, tabulated with costs. **Standards**: Compression ratio, file type, length (s), frame rate (fps), resolution of recording (pixels), resolution of viewing screen (pixels), file type used for transfer, mean bandwidth during file transfer (kbps or Mbps). **Image quality assessment**: Evidence of use of an appropriate method e.g. Simultaneous stimulus relative quality scale method [43]. **Statistics**: Use of appropriate statistical tests.	**Reliability**: Transmission stability: % recordings received as total of a) transmissions b) time, Packet loss, transmission delay (s), Jitter, Inter-rater and intra-rater reliability. **Objective image quality**: VIF, VSNR. **Diagnostic efficacy**: AUC, Sensitivity and specificity. **Clinical utility**: change to treatment plan, operative or medical treatment frequency, HRQOL or morbidity and mortality. **Cost effectiveness**: QALY. **Qualitative outcomes**: User opinions from survey or interviews.	**Internal validity**: Including comment on patient, index test, reference and setting. **Study limitations**: Must be included. **External validity**: Including the applicability to clinical settings.

BMI: Body mass index. Kbps: Kilobits Per Second. Mbps: Megabits Per Second. VIF: Visual Information Fidelity. VISNR: visual signal-to-noise ratio VAS: Visual analogue scale. AUC: Area under curve. HRQOL: Health Related Quality of Life. QALY: quality-adjusted life years.

#### Limitations of this review

This review has several limitations. First, the poor methodological quality of a significant proportion of the studies in this review has limited the conclusions that can be drawn. Second, the definition of suitable inclusion criteria was difficult. For example, the definition of an emergency setting is contextual. What may be an emergency in a remote island may not be an emergency in an inner city with plentiful healthcare resources. This may have led us to include or exclude potentially relevant articles. This also led to the decision to include articles in a general medicine context containing both chronic and emergent conditions. However, given the difficulties in identifying all articles that might contain evidence of the utility of telesonography in emergency circumstances this review should be considered in the context of reviews of telesonography in other fields of medicine. Additionally, despite the prevalence of cardiovascular disease in emergency medicine, we excluded studies that exclusively considered echocardiography. This decision was made because there are a large volume of specialist cardiology papers in which the relevance to emergency medicine was unclear, and thus may be more appropriate to evaluate separately. A review of tele-echocardiography is available from Frumento et al. [88]. Papers concerning neonatal medicine were excluded for similar reasons.

Last, given that the included studies are largely small and experimental in nature it is possible that there are many small feasibility studies where negative findings have gone unpublished. From a technological perspective the presence of such a publication bias may limit knowledge of the circumstances which prevent successful telesonography but does not affect the assertion that telesonography is feasible (which requires only a single successful demonstration). However, the likely presence of publication bias may have a more significant impact if there are a number of unpublished pilot studies that have found telesonography to be clinically redundant or harmful.

## Conclusions

Telesonography has been demonstrated to be feasible for use in emergency medicine. It has also been demonstrated to have comparable diagnostic power to conventional in-person ultrasonography. It has not however yet been demonstrated to be clinically effective. While the potential benefits have been described, particularly in the context of under-resourced settings, they have not been adequately demonstrated in practice and require more robust evidence before implementation. Future studies should make efforts to ensure that their methodology and results contain information which can be used to inform future study design, as further definitive study is required before telesonography can be wholeheartedly recommended for use in the emergency setting.

## Supporting information

S1 File Critical appraisal checklist adapted from the QUADAS criteria. (DOCX)

S1 Table Prisma 2009 checklist. (DOC)

S2 Table Search strategy. (DOCX)

S3 Table Data extraction form. (DOCX)

S4 Table Study methods. N: number of participants. NR: Not reported.
(DOCX)

S5 Table Critical appraisal results. Risk of Bias using the amended QUADAS criteria. Code 1 = low risk or concern 2 = uncertain risk or concern 3 = high risk or concern 4 = item not applicable.
(DOCX)

S6 Table Technical feasibility. (DOCX)
