# Supplementary material for: Telesonography in emergency medicine: A systematic review
Source: PLoS One. 2018 May 3;13(5):e0194840. doi: 10.1371/journal.pone.0194840 (PMC5933714; doi:10.1371/journal.pone.0194840)
Supplement: S1 File — (DOCX) [file pone.0194840.s001.docx]

**S1 File. Critical appraisal checklist, adapted from the QUADAS criteria.**

**Domain 1: Generic quality standards****

1. Does the study address an appropriate and clearly focused question? ****** *Yes/no/unclear*
2. Was the study design appropriate to meet study aims? ******

*Yes/no/unclear*

1. Was the study size appropriate to meet study aims?******

*Yes/no/unclear*

1. Was there evidence that the authors had NOT been influenced by vested interest?****** *Yes/no/unclear*
2. Confirmed ethical approval? ******

*Yes/no/unclear*

1. Did they take informed consent from all parties involved? Or appropriate waiver stated? ******

*Yes/no/unclear*

1. Were the methods described of sufficient detail to enable study replication? ****** *Yes/no/unclear*
2. Have they reported on all the outcomes stated within aims/methods?(is there a risk of selective reporting) ******

*Yes/no/unclear*

1. Did the authors report on study limitations? ******

*Yes/no/unclear*

1. Did the author report on generalisability/external validity? ******

*Yes/no/unclear*

**Domain 2: Patient/Participant selection:  Risk of bias**

1. Were the participants acutely unwell patients?******

*Yes/no/unclear/NA*

1. Were the simulated patients as representative as was feasible?******

*Yes/no/unclear/NA*

1. Was the level of expertise of reviewers and operators adequately described? ******

*Yes/no/unclear/NA*

1. Was an appropriate method of participant selection used?

*Yes/no/unclear*

1. Did the study avoid inappropriate exclusions of either participants or patients?

*Yes/no/unclear*

1. Could the selection of participants have introduced bias?

*RISK: High, low, unclear*

**Domain 3: Index test(s) (if more than 1 index test was used, please complete for each test:   Risk of bias**

1. Were the reviewers blinded to the health state of the patient? OR were they blinded to the mode of the transmission used? *****

*Yes/no/unclear/NA*

1. Were the outcome measures appropriate to the study aims? ******

*Yes/no/unclear/NA*

1. If subjective outcomes were reported, was an appropriate score or scale used eg. Likert scores? ******

*Yes/no/unclear/NA*

1. Were appropriate statistical tests used? ******

*Yes/no/unclear/NA*

1. Were statistics reported with confidence intervals? ******

*Yes/no/unclear/NA*

*Yes/no/unclear/NA*

1. Could the conduct or interpretation of the index test have introduced bias?

*RISK: High, low, unclear*

**Domain 4: Reference standard    Risk of bias**

1. Was a reference standard used?

*Yes/no/unclear/NA*

1. Is the reference standard likely to correctly classify the target condition?

*Yes/no/unclear/NA*

1. Were the reference standard results interpreted without knowledge of the results of the index test? (again were the participants/reviewers blinded to the comparison group outcomes)

*Yes/no/unclear/NA*

1. Could the reference standard, its conduct, or its interpretation have introduced bias?

*RISK: High, low, unclear*

**Domain 5: Flow and timing: Risk of bias**

1. Was there an appropriate interval between index test(s) and reference standard?

*Yes/no/unclear/NA*

1. If teaching outcomes were used was there an appropriate interval between teaching and assessment/ measurement? ******

*Yes/no/unclear/NA*

1. Did all patients receive a reference standard?

*Yes/no/unclear/NA*

1. Did patients receive the same reference standard?

*Yes/no/unclear/NA*

1. Were all patients/participants included in the analysis?

*Yes/no/unclear/NA*

1. Could the patient/participant flow have introduced bias?

*RISK: High, low, unclear*

**Domain 6: Telemedicine/ Feasibility specific concerns**

1. Was there reporting on the security measures relating to the transfer of images? ******

*Yes/no/unclear/NA*

1. Were the sending and/or receiving environments representative of clinical practice and/or the study aims? ******

*Yes/no/unclear/NA*

1. Were the communications and image/video resolution standards used reported? ******

*Yes/no/unclear/NA*

1. Was the level of cost described? ******

*Yes/no/unclear/NA*

1. Were technical barriers to implementation described? ******

*Yes/no/unclear/NA*

**Domain 7: Concerns regarding applicability**

1. Is there concern that the reference or comparison standard used does not match the review question?

*CONCERN: Low, high, unclear, NA*

1. Is there concern that the included patients do not match the review question? *CONCERN: Low, high, unclear, NA*
2. Is there concern that the mode of ultrasound, its conduct, or interpretation differ from the review question? ******

*CONCERN: Low, high, unclear, NA*

1. Is there concern that the mode of telecommunications used differ from the review question? ******

*CONCERN: Low, high, unclear, NA*

* Altered question

** New question
