# Supplementary material for: Telesonography in emergency medicine: A systematic review
Source: PLoS One. 2018 May 3;13(5):e0194840. doi: 10.1371/journal.pone.0194840 (PMC5933714; doi:10.1371/journal.pone.0194840)
Supplement: S2 Table — (DOCX) [file pone.0194840.s003.docx]

**S2 Table: Search Strategy**

| Database | Concept 1: Ultrasound | Concept 2: Telemedicine | Concept 3: Prehospital/remote |
| --- | --- | --- | --- |
| MEDLINE | 1. ultrasonic*.mp 2. ultrasound*.mp 3. sonograph*.mp 4. sonography.mp 5. telesonography*.mp 6. neurosonography.mp 7. ultrasonography/ ultrasonography, doppler/ ultrasonography 8. Ultrasonography, Interventional/is, mt [Instrumentation, Methods] 9. Echotomograph.mp 10. echocardiograph*.mp 11. Ultrasonics/ 12. ultraso*.mp 13. sonograph*.mp 14. fast scan.mp 15. transcranial ultrasound.mp | 1. remote consultation/ 2. remote consult*.mp 3. remote.mp 4. remotely supported.mp 5. remotely guided.mp 6. Emergency Service, Hospital/ 7. tele* 8. teleradiology.mp 9. Teleradiology/ 10. Telecommunications/ 11. "Referral and Consultation"/ 12. telemonitor* 13. telemedicine.mp 14. telemedicine/ 15. teleultrasound.mp 16. Videotape Recording/is, mt, ut [Instrumentation, Methods, Utilization] 17. videoconferencing.mp 18. Videoconferencing/ 19. Video Recording/ or video recording*.mp 20. cell phones.mp or Cell Phones/ 21. computer communication.mp 22. (networks and instrumentation).mp 23. Internet/is [Instrumentation] 24. Telecommunications/is [Instrumentation] 25. Satellite communication.mp 26. satellite communications/ | 1. emergency medical services/mt [Methods] 2. emergency medical service*.mp 3. austere environment mp. 4. emergency medic* 5. emergency treatment/is [Instrumentation] 6. first aid/is [Instrumentation] 7. Emergency Medical Technicians/ 8. ems.mp 9. emt.mp 10. military medicine/ 11. wilderness medicine/ 12. austere environment.mp 13. Military Personnel/di, ra [Diagnosis, Radiography] 14. Emergency Medical Technicians/ 15. emergency technician.mp 16. emergency practitioner 17. Primary health Care/ 18. General Practitioner/ 19. Nurse Practitioner/ 20. Family Practice/ 21. emergency care practitioner mp. 22. emergency dispatch.mp 23. emergency rescue.mp 24. emergency resus*.mp 25. Triage/emergency triage.mp 26. military medicine/ 27. ambulance*.mp 28. Ambulances 29. Air Ambulances/ 30. Aircraft/ 31. air ambulance*.mp 32. "Transportation of Patients"/ 33. Prehospital.mp 34. Time Factors/ 35. pre-hospital paramedic.mp 36. Allied Health Personnel/ 37. out-of-hospital.mp 38. out of hospital.mp 39. out-hospital.mp 40. Emergency Responders/ 41. first responder.mp 42. Disaster Planning/ |
| CINAHL | 1. Ultrasound 2. ultrasonic 3. ultrasonography 4. sonography 5. sonograph* 6. telesonography 7. telesonograph* 8. neurosonography 9. neurosonograph* 10. Duplex 11. duplex ultrasound 12. doppler 13. Doppler ultrasound 14. Doppler ultrasonography 15. echocardiograph* 16. echo 17. fast scan 18. transcranial ultrasound | 1. remote consultation 2. remote consult* 3. remote 4. remotely supported 5. telecommunication 6. telemedicine 7. telehealth 8. telecare 9. teleultrasound 10. Videorecording 11. videotaping 12. videoconferencing 13. videocalling 14. video calling 15. videoconference 16. cell phones 17. mobile phone 18. computer communication system 19. Satellite 20. satellite communications 21. (heading) Emergency medical service communications systems 22. (heading)Alternative and augmentive communication 23. networks and instrumentation 24. computer technology | 1. emergency medical services 2. EMS 3. emergency medical service* 4. emergency medicine 5. austere environment 6. emergency medic* 7. emergency treatment 8. first aid 9. Emergency Medical Technician 10. paramedic 11. wilderness medicine 12. wilderness management 13. austere environment 14. military medicine 15. military personnel 16. combat medicine 17. emergency practitioner 18. Primary health Care 19. Family Practice 20. Primary health Care 21. General Practice 22. General Practitioner 23. Nurse Practitioner 24. emergency care practitioner 25. emergency dispatch 26. emergency dispatcher 27. emergency rescue 28. emergency resuscitation 29. Triage 30. Air Ambulances 31. Transport of patients 32. [transportation of patients](http://web.a.ebscohost.com/ehost/breadbox/search?term=transportation%20of%20patients&sid=6877b46f-6026-4ddc-8a8c-e24356a5cd9d%40sessionmgr4007&vid=32) 33. prehospital 34. pre hospital 35. pre-hospital 36. out of hospital 37. rural 38. non hospital setting 39. emergency responder* 40. disaster response and emergency management |
| EMBASE | 1. ultrasonic*.mp 2. ultrasound.mp 3. portable ultrasound scanner/ 4. ultrasound/ 5. ultrasound scanner/ 6. diagnosis/ 7. echography/ 8. sonograph.mp 9. brain injury/ 10. ultrasound/ 11. echography/ 12. b scan/ 13. doppler ultrasonography/ 14. echocardiography/ echotomography/ 15. real time echography/ 16. telesonography*.mp 17. neurosonography.mp 18. ultrasonography.mp 19. echocardiography/ 20. Doppler echocardiography/ 21. echocardiograph/ 22. echocardiograph*.mp 23. ultrasonic.mp ultrasound/ 24. fast scan.mp 25. Doppler echography/ or transcranial ultrasound.mp 26. Doppler ultrasonography/ 27. transcranial doppler/ 28. Doppler echocardiography/ 29. duplex Doppler ultrasonography/ 30. pulsed Doppler ultrasonography/ 31. doppler echo/ 32. transcranial Doppler ultrasonography/ 33. laser Doppler flowmetry/ 34. Doppler flowmeter/ 35. color Doppler flowmetry/ 36. color Doppler echocardiography/ 37. doppler.mp 38. tissue Doppler imaging/ 39. Doppler flowmetry/ | 1. remote consultation.mp 2. teleconsultation/ 3. remotely supported.mp 4. telemedicine/ 5. remotely guided.mp 6. teleultrasound.mp or telecommunication/ 7. telecommunication/ 8. teleradiology/ 9. teleradiology.mp 10. videotape recording.mp 11. videorecording/ 12. videoconferencing.mp 13. videoconferencing/ 14. cell phones.mp 15. mobile phone/ 16. computer network/ 17. computer/ 18. computer program/ 19. computer communication.mp 20. Internet/ 21. telecommunication/ 22. telehealth/ | 1. emergency health service 2. emergency medical service*.mp 3. emergency care/ 4. emergency care practitioner.mp 5. emergency medicine/ 6. emergency medic.mp 7. army/ 8. military medicine/ 9. battle injury/ 10. austere environment.mp 11. war/ 12. soldier/ 13. prehospital.mp 14. first aid/ 15. emergency medical technicians.mp or rescue personnel/ 16. ems.mp 17. emt.mp 18. paramedical personnel/ 19. wilderness medicine.mp 20. wilderness medicine/ 21. austere environment.mp 22. emergency dispatch.mp 23. emergency resus*.mp 24. triage.mp 25. ambulance transportation/ 26. ambulance/ 27. ambulance*.mp 28. air medical transport/ 29. air ambulance*.mp 30. helicopter/ 31. Time Factors/ 32. out-of-hospital.mp 33. out of hospital.mp 34. out-of hospital.mp 35. out-hospital.mp 36. Emergency Responders.mp 37. first responder.mp 38. disaster planning./ 39. Primary health Care/ 40. general Practitioner/ 41. nurse Practitioner/ 42. General Practice/ |
| The Cochrane Library | 1. ultrasound.mp 2. sonography.mp 3. ultrasonography.mp 4. echography.mp 5. echocardiocardiography.mp 6. B mode.mp 7. Doppler.mp 8. Duplex.mp | 1. remote consultation.mp 2. teleconsultation.mp 3. telemedicine.mp 4. telehealth.mp 5. telecommunication.mp 6. teleradiology.mp 7. tele* 8. videotape recording.mp 9. videoconferencing.mp 10. cell phones.mp 11. mobile phone.mp 12. computer network.mp 13. computer program.mp 14. computer communication.mp 15. Internet.mp | 1. emergency health care.mp or 2. emergency care.mp or 3. emergency care practitioner.mp or 4. emergency medic.mp or 5. military medicine.mp or 6. prehospital.mp or 7. first aid.mp or 8. emergency medical technicians.mp or 9. ems.mp or 10. emt.mp or 11. paramedic or 12. paramedical personnel.mp or 13. wilderness medicine.mp or 14. community health worker or 15. austere environment.mp or 16. emergency dispatch.mp or 17. emergency resus*.mp or 18. triage.mp or 19. rural.mp or 20. ambulance.mp or 21. ambulance transportation.mp or 22. air medical transport.mp or 23. air ambulance*.mp or 24. helicopter.mp or 25. out-of-hospital.mp or 26. out of hospital.mp or 27. out-of hospital.mp or 28. out-hospital.mp or 29. Emergency Responders.mp or 30. first responder.mp or 31. disaster planning mp or 32. Primary health Care.mp or 33. Primary health Care.mp or 34. general Practitioner.mp or 35. nurse Practitioner.mp or 36. General Practice .mp |
