# Supplementary material for: Telesonography in emergency medicine: A systematic review
Source: PLoS One. 2018 May 3;13(5):e0194840. doi: 10.1371/journal.pone.0194840 (PMC5933714; doi:10.1371/journal.pone.0194840)
Supplement: S3 Table — (DOCX) [file pone.0194840.s004.docx]

**S3 Table. Data extraction form**

| 1: Data Extraction Details | |
| --- | --- |
| Reviewer ID: |  |
| Link to full text: |  |
| Date of data extraction: |  |
| Meets full inclusion/inclusion criteria requirements? |  |
| Decision, INCLUDE or EXCLUDE: |  |

| 1: INCLUSION EXCLUSION CRITERIA | | | Criteria met? |
| --- | --- | --- | --- |
| Domain | Inclusion | Exclusion |  |
| Study type: | 1. Design 2. Prospective observational studies 3. Case series 4. Feasibility study 5. Pilot study in clinical setting 6. Economic analysis 7. Randomised controlled trial   **AND**   1. Full text   **AND**   1. Published in a peer reviewed journal | 1. Design 2. Case studies 3. Conference proceeding 4. Abstracts 5. Posters   **OR**   1. Full text not available after request   **OR**   1. Unpublished trials |  |
| Participants: | 1. Patients 2. Human 3. Clinical setting 4. Acutely unwell patients 5. Simulated patients 6. Studies in which emergency cases were present but do not form the bulk of the patient population as long as the study outcomes and methods could be applied to an emergency setting.   **AND**   1. Professionals 2. Operators separate from image assessors | 1. Patients   1. Neonates  2. Infants  **OR**   1. Scans only concerned with echocardiography |  |
| Setting: | 1. Emergency medical department Prehospital care 2. Hospital wards 3. Remote and rural clinic providing medical for emergencies | 1. Urban general practice |  |
| Procedure: | 1. Any form of ultrasound scan, Sonographer and expert image reviewer in separate locations 2. Sonographer and/or reviewer involved in the provision of emergency medical services. 3. Scan of a region or for an indication directly relevant to the emergency services. 4. Scan for the purposes for detecting an urgent and serious pathology where there it is probable that the findings of scans would require same day change in clinical management. | 1. Scans acquired and interpreted by the same practitioner. 2. Sonographer and reviewer unlikely to be involved in the provision of emergency medical services. 3. Scans performed for indications that would not be performed in an emergency medical situation. 4. Scans in which it is highly unlikely that the outcome would require a same day change in clinical management. |  |
| Aims and Outcomes: | 1. Intention    1. Ultrasound with the potential to impact patient management    2. Ultrasound for use in an acute setting.    3. Preliminary management or patient transport as the anticipated outcome of ultrasound.    4. Aims relate to the provision of emergency medical care.   **AND**   1. Outcomes    1. Clinical utility of images    2. Image quality    3. Improvements to patient outcome | 1. Intention    1. Aims that are not relevant to telesonography in emergency medicine.   **AND**   1. Outcomes    1. No outcomes related to either the clinical usefulness of images.    2. No diagnostic outcome of images.    3. Studies in which under two patients required immediate or urgent care. |  |
| Other | 1. Date    1. 1946 to present   **AND**   1. Language    1. English,    2. French    3. German    4. Norwegian    5. Russian    6. Danish    7. Mandarin | 1. Date    1. Pre- 1946   **OR**   1. Language    1. Other languages |  |

| **STUDY INFORMATION:** | |
| --- | --- |
| **Domain 1: Study Characteristics** | |
| Study ID: |  |
| Study Title: |  |
| Study size: |  |
| Description of aim/study hypothesis: |  |
| Main study findings: |  |
| Evaluative summary: *(Main strengths and weaknesses of paper as noted by reviewer)* |  |
| **Domain 2: Study Methodology** | |
| Short description of methodology including comparison groups and timing: |  |
| Scan type: |  |
| Reference standard: |  |
| Transmission type: |  |
| Transmitting |  |
| Setting: |  |
| Operator: |  |
| Ultrasound equipment: |  |
| Other hardware/equipment: |  |
| Other software: |  |
| Telecommunication system used: |  |
| Receiving equipment: |  |
| Setting: |  |
| Receiver/Remote expert: |  |
| Viewing equipment: |  |
| Other equipment: |  |
| Training of participants: |  |
| Other pertinent system design details: |  |
| Specifications: |  |
| Spacial resolution of images: |  |
| Special resolution of viewing screen: |  |
| Temporal resolution/frame rate: |  |
| Communication bandwidth: |  |
| Other communication specifications reported: |  |
| **Domain 3: Key Results** |  |
| Proportion of successful scans performed: |  |
| Reliability/transmission failures: |  |
| Diagnostic accuracy (sensitivity/specificity/concordance): |  |
| Evidence of clinical utility: |  |
| Pathology, patients: |  |
| Number of enrolled participants: |  |
| Participant characteristics (age, BMI): |  |
| Clinical findings: |  |
| Scan duration: |  |
| Transmission time/delay: |  |
| User attitudes: |  |
| **Domain 4: Author Interpretation** | |
| Summary of main findings: |  |
| Conclusion: |  |
| Study Limitations: |  |
| Pertinent discussion points: |  |

**REVISED CRITICAL APPRAISAL CHECKLIST**

| **Domain 1: Generic quality standards** | **Decision** |
| --- | --- |
| 1. Does the study address an appropriate and clearly focused question? | Yes/no/unclear |
| 1. Was the study design appropriate to meet study aims? | Yes/no/unclear |
| 1. Was the study size appropriate? | Yes/no/unclear |
| 1. Was there evidence that the authors had NOT been influenced by vested interest? | Yes/no/unclear |
| 1. Confirmed ethical approval? | Yes/no/unclear |
| 1. Did they take informed consent from all parties involved? Or appropriate waiver stated? | Yes/no/unclear |
| 1. Were the methods described of sufficient detail to enable study replication? | Yes/no/unclear |
| 1. Have they reported on all the outcomes stated within aims/methods? (is there a risk of selective reporting) | Yes/no/unclear |
| 1. Did the authors report on study limitations? | Yes/no/unclear |
| 1. Did the author report on generalisability/external validity? | Yes/no/unclear |
| **Domain 2: Patient/Participant selection** |  |
| 1. Were the participants acutely unwell patients? | Yes/no/unclear/NA |
| 1. Were the simulated patients as representative as was feasible? | Yes/no/unclear/NA |
| 1. Was the level of expertise of reviewers and operators adequately described? | Yes/no/unclear/NA |
| 1. Was an appropriate method of participant selection used? | Yes/no/unclear/NA |
| 1. Did the study avoid inappropriate exclusions of either participants or patients? | Yes/no/unclear/NA |
| 1. Could the selection of participants have introduced bias? | RISK: High, low, unclear |
| **Domain 3: Index test(s)** |  |
| 1. Were the reviewers blinded to the health state of the patient? OR were they blinded to the mode of the transmission used? | Yes/no/unclear/NA |
| 1. Were the outcome measures appropriate to the study aims? | Yes/no/unclear/NA |
| 1. If scoring systems were used were they appropriate (Likert or equivalent with relevant categories? | Yes/no/unclear/NA |
| 1. Were appropriate statistical tests used? | Yes/no/unclear/NA |
| 1. Were statistics reported with confidence intervals? | Yes/no/unclear/NA |
| 1. If subjective outcome measures were used, was it unlikely that questions introduce bias within the phrasing? | Yes/no/unclear/NA |
| 1. Could the conduct or interpretation of the index test have introduced bias? | Yes/no/unclear/NA |
| **Domain 4: Reference standard** |  |
| 1. Was a reference standard used? | Yes/no/unclear/NA |
| 1. Is the reference standard likely to correctly classify the target condition? | Yes/no/unclear/NA |
| 1. Were the reference standard results interpreted without knowledge of the results of the index test? (again were the participants/reviewers blinded to the comparison group outcomes) | Yes/no/unclear/NA |
| 1. Could the reference standard, its conduct, or its interpretation have introduced bias? | RISK: High, low, unclear |
| **Domain 5: Flow and timing** |  |
| 1. Was there an appropriate interval between index test(s) and reference standard? | Yes/no/unclear/NA |
| 1. If teaching outcomes were used was there an appropriate interval between teaching and assessment/ measurement? | Yes/no/unclear/NA |
| 1. Did all patients receive a reference standard? | Yes/no/unclear/NA |
| 1. Did patients receive the same reference standard? | Yes/no/unclear/NA |
| 1. Were all patients/participants included in the analysis? | Yes/no/unclear/NA |
| 1. Could the patient/participant flow have introduced bias? | RISK: High, low, unclear |
| **Domain 6: Telemedicine/ Feasibility specific concerns** |  |
| 1. Was there reporting on the security measures relating to the transfer of images? | Yes/no/unclear/NA |
| 1. Were the sending and/or receiving environments representative of clinical practice and/or the study aims? | Yes/no/unclear/NA |
| 1. Were the communications and image/video resolution standards used reported? | Yes/no/unclear/NA |
| 1. Was the communications system used widely available? | Yes/no/unclear/NA |
| 1. Was the level of cost described? | Yes/no/unclear/NA |
| 1. Were technical barriers to implementation described? | Yes/no/unclear/NA |
| **Domain 7: Concerns regarding applicability** |  |
| 1. Is there concern that the reference or comparison standard used does not match the review question? | CONCERN: Low, high, unclear, NA |
| 1. Is there concern that the included patients do not match the review question? | CONCERN: Low, high, unclear, NA |
| 1. Is there concern that the mode of ultrasound, its conduct, or interpretation differ from the review question? | CONCERN: Low, high, unclear, NA |
| 1. Is there concern that the mode of telecommunications used differ from the review question? | CONCERN: Low, high, unclear, NA |
| 1. Is there concern that the operators were not representative of typical practitioners in emergency departments or prehospital care? | CONCERN: Low, high, unclear, NA |
