# Supplementary material for: Telesonography in emergency medicine: A systematic review
Source: PLoS One. 2018 May 3;13(5):e0194840. doi: 10.1371/journal.pone.0194840 (PMC5933714; doi:10.1371/journal.pone.0194840)
Supplement: S4 Table — N: number of participants. NR: Not reported. (DOCX) [file pone.0194840.s005.docx]

**S4 Table: Study methods**

| Study ID | Description of design | Operating environment | Receiving environment | Technical details |
| --- | --- | --- | --- | --- |
| Adambounou 2014 | Pilot study evaluating telesonography system linking peripheral hospitals & University hospitals. Asynchronous and Synchronous transmission of videos and static images. Range of views, emergent and non-emergent pathologies. | **Environment:** Patient centre in Togo  **Operator**: Junior sonographer/ radiologist or lay operator (e.g., midwife, nurse, technician)  **Training:** No specific training given  **Technology:** Network camera, Internet video server, Skype, video converter/USB, 3G dongle  **N**=50 (patients) | **Communications:** ASDL, fibreoptic & 3G  **Environment:** Lomé University Hospital  **Receiver:** Radiologist  **Technology:** Workstation, 3D imaging platform, remote access software, free online internet speed checker | **Frame rate:** 10fps- 35fps  **Resolution:** NR  **Bandwidth:** 1 Mbps (IP Camera) average 512 kbps (remote access software)  **Transmission delay:** ≈ 1.5s |
| Adhikari 2014 | Simulated experiment to examine possibility of real time transmission ultrasound images from a disaster zone. Synchronous transmission of videos and static images. FAST scans of healthy volunteers. | **Environment**: Simulated disaster scene, car park USA.  **Operator:** 1 emergency physician  **Training:** No specific training  **Technology:** Portable ultrasound system, camera phone. Cellular transmission via mobile phone  **N**=19 (videos) N=1 (operators) N=3 (reviewers) | **Communications:** Cellular transmission  **Environment:** Emergency department  **Receiver:** 3 emergency physicians  **Technology:** Mobile phone | **Frame rate:** NR  **Resolution:** 176x144pixels  **Bandwidth:** NR  **Transmission delay:** median 82.5 seconds (95% CI, 67.7 seconds-97.3 seconds) |
| Al-Kadi 2009 | User survey concerning system of telesonography for resuscitation linking remote hospital and large trauma centre. Synchronous transmission of videos from FAST & EFAST scans. | **Environment**: Community Hospital Canada  **Operator:** Range of medical staff  **Training:** No specific training  **Technology:** Ultrasound machine, dedicated VC system  **N**=23 (patients) N=14 (users surveyed) | **Communications:** VC link (constrained to simulate satellite link)  **Environment:** Referral centre ED  **Receiver:** 2 trauma surgeons, 1 emergency physician  **Technology:** Standard monitors & VC system | **Frame rate:** NR  **Resolution:** NR  **Bandwidth:** NR  **Transmission delay:** NR |
| Biegler 2013 | Feasibility of telementoring nurse practitioners to assess for pneumothoraces in patients after chest tube removal. Synchronous transmission of videos of the pleural space. | **Environment**: Tertiary Care Hospital Canada  **Operator:** 1 nurse practitioner, 1 registered nurse  **Training:** No specific training  **Technology:** Ultrasound machine, head-camera, analogue to digital video converter, CODEC, Skype VC  **N=**13 (patients) N=2 (nurses) N= 1 (physician mentor) | **Communications:** Fixed LAN & Wi-fi  **Environment:** Personal home & hotel  **Receiver:** Trauma surgeon  **Technology:** Laptop, graphical user interface software | **Frame rate:** NR  **Resolution:** NR  **Bandwidth:** NR  **Transmission delay:** NR |
| Blaivas 2004 | Comparison between the images from a thermal printer to the images sent between two camera equipped mobile phones. Asynchronous transmission of still images of range of pathologies. | **Environment**: Trauma centre Emergency department  **Operator:** NA (photographs of pre-recorded images from ED)  **Training:** No specific training  **Technology:** Ultrasound machines, Mobile phone camera  **N=**50 (thermal prints photographed) N=2 (reviewers) | **Communications:** Cellular connection  **Environment:** Emergency department  **Receiver:** Emergency sonologist  **Technology:** Mobile phone | **Frame rate:** NA (stills)  **Resolution:** 640x480pixels  **Bandwidth:** 14kbytes/s (upload) 9kbytes/s (download)  **Transmission delay:** NR |
| Boniface 2011 | Feasibility of telementoring paramedics to conduct FAST exams on simulated patients. Synchronous transmission of videos. | **Environment**: Emergency medicine department, USA  **Operator:** Paramedics  **Training:** 20 mins Didactic training, cue card prompts  **Technology:** Ultrasound machine, 2-way radio  **N=**1 (model patient) N=51 (paramedics) N=2 (emergency physicians) | **Communications:** Cable video feed  **Environment:** Another room in same facility  **Receiver:** Emergency physician  **Technology:** Standard Monitor | **Frame rate:** NR  **Resolution:** NR  **Bandwidth:** NR  **Transmission delay:** NR |
| Courreges 2005 | Comparison of diagnostic agreement using the OTELO robotic system to remotely assess patient with conventional ultrasound scanning. Synchronous transmission of videos of heart and intraabdominal organs. | **Environment**: 1 Hospital in France 1 clinic in Spain  **Operator:** Robotic tele-ultrasound system (OTELO)  **Training:** No specific training given  **Technology:** (OTELO), laptop, VC camera  **N=**52 (patients) N=1 (probe holder) N=1 (robot operator) | **Communications:** ISDN lines, 3G mobile & Satellite transmission  **Environment:** Expert centre  **Receiver:** Robot operator/ultrasound expert  **Technology:** Robot controls, data storage, image server, videoconferencing camera | **Frame rate:** NR  **Resolution:** NR  **Bandwidth:** 1 x 128 kbps +1x64kbps line + 1x284 kbps satellite  **Transmission delay:** NR |
| Dyer 2008 | Findings from pilot study looking at telesonography for resuscitation linking remote hospital and large trauma centre Synchronous transmission of videos of FAST & EFAST scans. | **Environment**: Community hospital in Canada  **Operator:** Physicians, trainees and students  **Training:** No specific training given.  **Technology:** Ultrasound machine, high definition cameras, conference phone  **N=**20 (patients) N=18 (staff) | **Communications:** Unspecified internet connection. (designed with limitations to mimic satellite)  **Environment:** Large trauma hospital Canada  **Receiver:** 1 ER physician, 2 trauma surgeons and 1 neurologist  **Technology:** TFT monitor & LCD video monitor | **Frame rate:** NR  **Resolution:** NR  **Bandwidth:** 2Mbps  **Transmission delay:** 1-2s |
| Ito 2013 | Evaluation of the usability and performance of a wearable tele-sonography robot to be used by a paramedic in an ambulance. and Synchronous transmission of videos of FAST scans. | **Environment**: NR, Japan  **Operator:** Robotic Fast system  **Training:** No specific training  **Technology:** Portable ultrasound machine, wearable robot, videocamera, computer, router, speaker, CODEC  **N**=13 (healthy volunteers) N=9 (novices) N=1 (reviewer) | **Communications:** ISDN LAN (used for ultrasound video transmission), WIMAX  **Environment:** Hospital, Japan  **Receiver:** Emergency physician  **Technology:** Computer, router, microphone, CODEC | **Frame rate:** 25-30 fps  **Resolution:** NR  **Bandwidth:** ISDN (typically 128 kbps), LAN, and WiMAX (download: 40 Mbps, upload: 10 Mbps).  **Transmission delay:** <30ms |
| Johnson 1998 | A prospective study comparing diagnostic and management outcomes in two groups: A) on site radiologist support of trained sonographer compared to B) remote support of trained sonographer. A variety of scans. Asynchronous transmission of images and synchronous videoconferencing if deemed necessary. | **Environment**: Remote region in Alberta, Canada  **Operator:** Trained sonographer  **Training:** No specific training  **Technology:** Ultrasound machine, multiformat video, videoconferencing software, modem, telephone and computer  **N**=24 (emergency or urgent cases) N=146 (cases in total including elective and semi-urgent cases) N=1 (sonographer) N=4 (reviewers) | **Communications:** ½ T1 (telephone line)  **Environment:** University of Alberta  **Receiver:** Radiologist  **Technology:** Computer, modem, telephone | **Frame rate:** NR  **Resolution:** NR  **Bandwidth:** 19.2 kbps  **Transmission delay:** 5 mins for complete assessment transfer, real time VC also used |
| Kim 2015 | Comparison of telementored ultrasonography with: A) conventional resident performed scans and B) conventional attending performed scans for diagnosing acute appendicitis in paediatric patients. Synchronous transmission of videos to smartphone. | **Environment**: Academic emergency medicine department, Republic of Korea  **Operator:** Emergency medicine residents  **Training:** No specific training  **Technology:** Ultrasound machine with inbuilt transmission system, broadband server, IP camera, and headset  **N=** 115 (patients) N=12 (residents) N=3 (attending physicians) | **Communications:** Broadband fixed-line internet network & LTE internet network.  **Environment:** Emergency department  **Receiver:** 3 attending emergency medicine physicians  **Technology:** Smartphone, remote viewing application | **Frame rate:** 25 fps  **Resolution:** 880 x 660 pixels (100-150kB)  **Bandwidth:** mean 59.1Mbps (±13.8)  **Transmission delay:** NR |
| Kim 2016 | Comparison of videos viewed using smartphone vs those viewed using LCD screen done for A) normal vs abnormal adult echocardiograms B) normal vs abnormal scans for paediatric appendicitis. Asynchronous and Synchronous transmission of videos and images. | **Environment**: Academic emergency medicine department, Republic of Korea  **Operator:** Emergency physician  **Training:** No specific training  **Technology:** Ultrasound machine with inbuilt transmission system, broadband server  **N=**200 (patients) N=12 (reviewers) | **Communications:** Broadband fixed-line internet network & 3G mobile network.  **Environment:** Emergency department  **Receiver:** 12 emergency physicians  **Technology:** LCD monitor, smartphone | **Frame rate:** 15 fps (echo) 4fps (appendix)  **Resolution:** 1366x 768 pixels (LCD)1136 x 640 pixels (smartphone) (echo 40 KB) (appendix 150kB (110-140))  **Bandwidth:** 3G=5.54 Mbps  **Transmission delay:** NR |
| Kwon 2015 | Feasibility of using training methods and scanning protocols from NASA to allow athletic trainers to obtain and transmit diagnostic images of musculoskeletal injuries in athletes. Synchronous transmission of videos of focused MSK ultrasounds. | **Environment**: Ice rinks  **Operator:** Athletic trainers  **Training:** 2 hrs didactic training, computer based proficiency tests and cue cards  **Technology:** Portable ultrasound machine, USB internet device, phone  **N=**32 (patients)N=4 (ultrasound operators) N=1(telementor) | **Communications:** Phone line & Internet  **Environment:** Hospital USA  **Receiver:** Physicians  **Technology:** NR | **Frame rate:** NR  **Resolution:** NR  **Bandwidth:** NR  **Transmission delay:** NR |
| Kolbe 2015 | Pilot study to examine use of didactic in person teaching as well as telementoring of point of care ultrasound to train staff and diagnose patients in a remote location in rural Nicaragua. Synchronous transmission of videos for a variety of indications. | **Environment**: Rural community Nicaragua  **Operator:** Local physicians and nurses  **Training:** Didactic teaching and workshops. Instructors had once weekly remote training sessions with staff  **Technology:** Ultrasound and laptops  **N=** 132 (patients) N=4 (Nicaraguan staff) | **Communications:** NR  **Environment:** USA  **Receiver:** Ultrasound experts  **Technology:** Computer, remote viewing/framegrabbing software, Skype | **Frame rate:** NR  **Resolution:** NR  **Bandwidth:** NR  **Transmission delay:** NR |
| Lee 2016 | Randomised crossover study, comparing remote mentoring to onsite mentoring to acquire ultrasound images of the appendix on simulated patients. Synchronous transmission of videos and static images. | **Environment**: Emergency department, Republic of Korea  **Operator:** 30 volunteers without USS experience  **Training:** 2h lecture and hands on workshop  **Technology:** Ultrasound machine with inbuilt transmission system, IP camera, computer server  **N**=10 (simulated patients) N=30 (volunteer operators) N=3 (reviewers) N=90 (scans) | **Communications:** Remote smartphone over LTE network  **Environment:** NR  **Receiver:** Emergency physicians  **Technology:** Smartphone, Remote viewing application | **Frame rate:** 25fps  **Resolution:** 1136 x 640  **Bandwidth:** 55.5 Mbps (± 8.6).  **Transmission delay:** 150 kB in PNG format 1136 × 640 (326 PPI) |
| Levine 2015 | Comparison ultrasound videos obtained from telementored ultrasound scans using high end tele-ICU system vs conventional scanning carried out by experts. Synchronous transmission plus asynchronous over-reading of screenshotted images. Range of views: internal jugular, right upper lobe, left upper lobe, right lung base, left lung base, subxiphoid, and bladder. | **Environment**: Simulated ICU room, USA  **Operator:** Various non-physician medical providers  **Training:** 20 min didactic training for operators and e-learning videos  **Technology:** Ultrasound machine, wall mounted camera, tele-ICU system  **N**=1 (simulated patient) N=1 (telementor) N=11 (non-physician ultrasound operators) | **Communications:** NR (centralised ICU system)  **Environment:** NR  **Receiver:** Emergency physician & independent ultrasound expert.  **Technology:** Computer and VISICU viewing system | **Frame rate:** NR  **Resolution:** 380k pixel tele-ICU camera  **Bandwidth:** NR  **Transmission delay:** NR |
| Levine 2016 | Comparison of ultrasound videos obtained from telementored ultrasound scans using Facetime vs images acquired directly from the ultrasound machine. Synchronous transmission of videos. Range of views: internal jugular, right upper lobe, left upper lobe, right lung base, left lung base, subxiphoid, and bladder. | **Environment**: Simulated ICU room, USA  **Operator:** 11 non-physician medical staff members  **Training:** No specific training  **Technology:** Ultrasound machine, wall mounted camera, tele-ICU system, iPads, Facetime  **N=**1 (healthy adult male patient) N=1 (telementor) N=11 (non-physician ultrasound operators) n=77 (images captured via facetime) N=1 (pilot study volunteer controlling the facetime images) | **Communications:** Wireless connection using router  **Environment:** Intensive care room  **Receiver:** Intensive care physician.  **Technology:** Laptop and VISICU viewing system | **Frame rate:** NR  **Resolution:** 720-pixel on iPad  **Bandwidth:** NR  **Transmission delay:** NR |
| Litelpo 2010 | Feasibility of trans-Atlantic transmission of pre-recorded ultrasound videos to an iPhone for the interpretation of a remote expert with real time interaction of transmitting and receiving physician. Synchronous transmission of videos and static images. Range of views and pathologies. | **Environment**: Boston US  **Operator:** Scanning physician  **Training:** No specific training  **Technology:** Ultrasound machine, digital video converter, S cable, laptop, fire wire  **N=**8 video clips N=1 (scanning physician) N=1 (interpreting physician) | **Communications:** Wi-Fi, 3G  **Environment:** Oxford UK  **Receiver:** Over-read by 8 emergency medicine physicians.  **Technology:** Phone, skype, call recorders for IChat and skype, Wi-fi router | **Frame rate:**Wi-Fi (1.1 fps) 3G (0.4 fps).  **Resolution:**  720 x 480 pixels  **Bandwidth:**  140 kbps (range 120–160 kbps)  **Transmission delay:** 2.7 s |
| Litelpo 2011 | Comparison of 4 different methods of transmitting pre-recorded ultrasound studies with real time interaction of transmitting and receiving physician. With overreading by blinded reviewers. Asynchronous and Synchronous transmission of videos and static images. Range of views and pathologies. | **Environment**: Boston US  **Operator**: Emergency physician  **Training:** No specific training  **Technology**: Ultrasound machine, digital video converter, S cable, laptop, fire wire, iChat and Skype**,** laptop, fire wire  **N**=5 (video clips) N=1 (scanning physician) N=8 (reviewing physicians) N=160 assessments. | **Communications:** 3G &Wi-Fi  **Environment:** Oxford UK  **Receiver:** Interpreting Physician  **Technology:** Laptop, call recorder software | **Frame rate:** 19.9-20.1fps (iChat, Wi-Fi)  14.8-19.9fps (Skype, Wi-Fi) 8.6-14.9 (Skype, 3G)  **Resolution:** 320x240 pixels. (iChat) 640x480 pixels (skype)  **Bandwidth:** 79.6-81.2 kBps (iChat, Wi-Fi)  50.7- 76.3 kbps (Skype, Wi-Fi) 10.6-18.7 kB/s (Skype, 3G)  **Transmission delay:** NR |
| Macedonia 1998 | Findings from pilot study use 3D telepresence and telesonography system for all pathologies linking remote military hospital and large expert centre. Asynchronous and telementored synchronous transmission of videos and static images including volumetric scans. | **Environment**: Mobile Army Surgical Hospital Bosnia  **Operator:** Ultrasound-trained physicians  and surgeons, ultrasound-naïve physicians and surgeons, nurses and paramedics  **Training:** Non-specific training  **Technology:** Portable ultrasound machine, telepresence ultrasound units, telephone  **N=**72 volumetric scans | **Communications:** ISDN and Ethernet CHECK  **Environment:** German hospital  **Receiver:** Remote consultants  **Technology:** Standard monitor, Immersion probe, telephone, scan converter, microphone, CDROM, zipdrive, videocapture. | **Frame rate:** 24fps  **Resolution:** 256x256 pixel, 4-20MByte  **Bandwidth:** Communications links from 9.6 to 1500kbit/s were used.  **Transmission delay:** 1-228minutes |
| McBeth 2011 | Findings from pilot study using expert telementoring to allow novice acquisition of Lung sliding data and FAST scans in various settings on simulated patients. Synchronous transmission of videos of EFAST and FAST scans. | **Environment**: On plane, in cafeteria, Lake Alberta, Banff Canada, Calgary Canada  **Operator:** 2 General surgeon trainees, emergency physician, banker, 7 yr old, 6 yr old  **Training:** No specific training  **Technology:** Portable ultrasound machine, analogue to digital converter, laptop, webcam, USB headset, smartphone  **N=**8 (patients) N=8 (novices) N=4 (supervisors) | **Communications:** 3G network  **Environment:** Residential homes & workplaces  **Receiver:** Emergency physician, general physician,  **Technology:** Laptop, Skype, remote viewing software | **Frame rate:** 15-10 fps  **Resolution:**  320 x240 pixels to 640 x480 pixels  **Bandwidth:** 600 Kbps to 1,400 Kbps (download) 500 Kbps and 800 Kbps (upload)  **Transmission delay:** NR |
| McBeth 2013 | Findings from pilot study looking at expert telementoring to allow novice acquisition of scans in various settings on simulated patients. Synchronous transmission of videos. FAST, focused Morrison’s pouch exam and EFAST. | **Environment**: Ambulance station, mountain first aid station and private home  **Operator:** 5 Emergency medical technicians, 10 ski patrollers, 4 nurses  **Training:** No specific training  **Technology:** Portable ultrasound machine, headset, webcam, laptop, 3G dongle, analogue to digital converter  **N=**2 (patients/phantom) N=19 (novices) N=7 (reviewers) N=1 (mentor) | **Communications:**  3G network  **Environment:** Home office, Café, pub  **Receiver:** 1 Physician mentor, over-read by 7 blinded physicians)  **Technology:** Skype, smartphone and laptop, remote viewing software | **Frame rate:** NR  **Resolution:** NR  **Bandwidth:** NR  **Transmission delay:** NR |
| Mikulik 2005 | Comparison of telementored transcranial Doppler and carotid duplex scans by a novice operator with those performed by expert neurosonographer. In healthy patients and those with recent stroke. Synchronous transmission of videos. | **Environment**: Neurology department  **Operator:** 3 healthcare providers novice to ultrasound, stroke nurse, neurologist and ophthalmologist  **Training:** No specific training  **Technology:** Portable ultrasound machine, camera, dual image screen  **N**=8 (patients) N=3 (novice operators) N=4 (remote experts) | **Communications:** High speed fibre-optic cable connection between rooms.  **Environment:** Neurology department  **Receiver:** Sonographers (1 acted as telementor, 1 blind over-read)  **Technology:** 2 monitors (1 for background another for ultrasound), camera, touchpad, hardware for remote viewing of videos | **Frame rate:** NR  **Resolution:** 800x 600 pixels  **Bandwidth:** NR  **Transmission delay:** NR |
| Nikolic 2006 | Feasibility of training junior ship officers to obtain diagnostic ultrasound images and transmit these to a remote expert. Asynchronous transmission of images, abdominal scans. | **Environment**: Faculty of Maritime Studies in Rijeka  **Operator:** Maritime officer trainees  **Training:** 90 mins didactic training, 30 mins hands on practice  **Technology:** Ultrasound machine, computer  **N=**3 (patients) N=37 (student operators) N=1 (reviewer) | **Communications:** NR  **Environment:** NR  **Receiver:** General surgeon  **Technology:** Computer, email | **Frame rate:** NA  **Resolution:** NR  **Bandwidth:** NR  **Transmission delay:** NR |
| Sibert 2008 | Feasibility of an ultrasound technician acquiring and transmitting diagnostically useful ultrasound videos from a simulated patient on a moving ambulance to physicians in the trauma center. Synchronous transmission of videos of carotid arteries and abdominal aorta. | **Environment**: Moving ambulance, USA  **Operator:** Ultrasound Technician  **Training:** No specific training  **Technology:** Ultrasound machine, overhead cameras, telemedicine software, monitor  **N=**1 (patient) N=9 (emergency trauma physicians) N=1 (ultrasound technician) | **Communications:** 3G with 6 cellular channels  **Environment:** Hospital  **Receiver:** Emergency  **Technology:** Monitor, telemedicine software. | **Frame rate:** NR  **Resolution:** NR  **Bandwidth:** 50–70 kbps  **Transmission delay:** NR |
| Song 2013 | Feasibility of a trained EMT acquiring and transmitting diagnostically useful FAST videos from a simulated patient on a moving ambulance to physicians in the emergency department. Synchronous and asynchronous transmission of images from 2 phantoms. | **Environment**: Moving ambulance, Korea  **Operator:** 1 EMT (with training in performing FAST**)**  **Training:** No specific training  **Technology:** Ultrasound machine**,** laptop, smartphone, framegrabber, computer, custom designed data handling software, viewer program, remote server  **N**=2 patients (1 phantom, 1 healthy volunteer) N=13 (emergency physicians) N=1 (EMT) | **Communications:** 3G & LAN  **Environment:** Emergency department  **Receiver:** 13 emergency physicians  **Technology:** Computer | **Frame rate:** NR  **Resolution:**  640x480 pixel  **Bandwidth:** NR  **Transmission delay**: NR |
| Strode 2003 | Comparison of effectiveness of transmission of scans using 4 modes of communication 1) Vest mounted microwave transmitter 2) Satellite 3) Life Link transmission system. Asynchronous transmission of FAST videos of patients with ascites, effusions and controls. | **Environment**: US Army Combat support Hospital  **Operator:** Sonologist  **Training:** No specific training  **Technology:** Ultrasound machine  **N=**5 (patients) N=1 (sonologist) N=15 (reviewers) | **Communications:** 2 different satellites**,** Lifelink and high gain sector antenna.  **Environment:** Emergency department, tertiary care centre  **Receiver:** 5 emergency physicians  **Technology:** laptop computer, power point. | **Frame rate:** 15-30fps (Lifelink)  **Resolution:** NR  **Bandwidth:** INMARSAT bandwidth 64kbps VSAT bandwidth 512 kbps  **Transmission delay:** NR |
| Zennaro 2016 | Comparison of diagnostic accuracy of point of care ultrasound when performed by telementored paediatricians vs onsite radiologists and blinded onsite radiologists. Synchronous transmission of videos. Paediatric lung, abdominal and hip scans. | **Environment**: Third level paediatric teaching hospital  **Operator:** Paediatrician  **Training:** 2h training course on ultrasound and 30 minutes teaching on tele-ultrasound system.  **Technology:** Ultrasound machine, web camera, iPod, microphone, encoder, headset, remote viewing application  **N=**52(patients) N=170 (ultrasound findings) | **Communications:** Wi-fi  **Environment:** Radiology department same hospital  **Receiver:** Radiologist  **Technology:** Laptop, microphone, remote viewing application. | **Frame rate:** NR  **Resolution:** NR  **Bandwidth:** NR  **Transmission delay:** 50ms (preset max. delay) |
