# Supplementary material for: Telesonography in emergency medicine: A systematic review
Source: PLoS One. 2018 May 3;13(5):e0194840. doi: 10.1371/journal.pone.0194840 (PMC5933714; doi:10.1371/journal.pone.0194840)
Supplement: S5 Table — Risk of Bias using the amended QUADAS criteria. Code 1 = low risk or concern 2 = uncertain risk or concern 3 = high risk or concern 4 = item not applicable. (DOCX) [file pone.0194840.s006.docx]

**S5 Table. Critical appraisal results.**

| **Domain 1: Generic quality standards** | | | | | | | | | | | | | | | | | | | | | | | | | | | | |
| --- | --- | --- | --- | --- | --- | --- | --- | --- | --- | --- | --- | --- | --- | --- | --- | --- | --- | --- | --- | --- | --- | --- | --- | --- | --- | --- | --- | --- |
|  | Adambounou 2014 | Adhikari 2014 | Alkadi 2009 | Biegler 2013 | Blaivas 2009 | Boniface 2011 | Courreges 2005 | Dyer 2008 | Ito 2013 | Johnson 1998 | Kim 2015 | Kim 2016 | Kolbe 2015 | Kwon 2007 | Lee 2016 | Levine 2015 | Levine 2016 | Litelpo 2010 | Litelpo 2011 | Macedonia 2008 | McBeth 2011 | McBeth 2013 | Mikulik 2005 | Nikolic 2006 | Sibert 2008 | Song 2013 | Strode 2003 | Zennaro 2016 |
| 1. *Focused question?* | 3 | 1 | 1 | 1 | 1 | 1 | 2 | 1 | 1 | 1 | 1 | 1 | 1 | 2 | 1 | 1 | 1 | 2 | 1 | 3 | 2 | 2 | 1 | 2 | 1 | 1 | 1 | 1 |
| 1. *Study design appropriate?* | 2 | 2 | 1 | 1 | 1 | 3 | 2 | 1 | 2 | 1 | 1 | 1 | 1 | 2 | 1 | 1 | 1 | 2 | 1 | 2 | 2 | 2 | 1 | 2 | 2 | 2 | 1 | 1 |
| 1. *Size appropriate?* | 2 | 1 | 1 | 1 | 1 | 2 | 1 | 1 | 3 | 1 | 1 | 1 | 1 | 1 | 1 | 1 | 1 | 2 | 1 | 1 | 2 | 1 | 1 | 1 | 2 | 2 | 1 | 1 |
| 1. *Conflict of interest* | 1 | 1 | 3 | 1 | 3 | 3 | 2 | 3 | 1 | 2 | 2 | 1 | 1 | 2 | 1 | 1 | 1 | 1 | 2 | 2 | 2 | 1 | 1 | 3 | 2 | 2 | 2 | 1 |
| 1. *Ethical approval* | 3 | 1 | 1 | 1 | 1 | 1 | 1 | 1 | 1 | 2 | 1 | 1 | 1 | 1 | 1 | 1 | 1 | 2 | 2 | 2 | 2 | 1 | 1 | 1 | 1 | 1 | 1 | 1 |
| 1. *Informed consent/waiver* | 1 | 1 | 2 | 1 | 2 | 1 | 1 | 2 | 1 | 1 | 1 | 1 | 1 | 1 | 1 | 1 | 2 | 2 | 2 | 1 | 2 | 3 | 1 | 3 | 2 | 2 | 1 | 1 |
| 1. *Replicable methods* | 3 | 2 | 2 | 1 | 1 | 2 | 3 | 1 | 2 | 1 | 2 | 1 | 2 | 1 | 1 | 1 | 1 | 3 | 3 | 3 | 1 | 1 | 1 | 2 | 3 | 3 | 1 | 1 |
| 1. *Selective reporting* | 2 | 1 | 1 | 1 | 3 | 1 | 3 | 1 | 1 | 1 | 1 | 1 | 1 | 1 | 1 | 1 | 1 | 2 | 1 | 2 | 3 | 1 | 1 | 1 | 1 | 1 | 1 | 1 |
| 1. *Study limitations?* | 1 | 1 | 1 | 1 | 1 | 1 | 3 | 1 | 3 | 1 | 1 | 3 | 3 | 3 | 3 | 1 | 1 | 1 | 1 | 1 | 3 | 1 | 1 | 1 | 3 | 1 | 1 | 1 |
| 1. *Generalisability reported?* | 1 | 1 | 3 | 1 | 1 | 1 | 3 | 1 | 1 | 2 | 1 | 1 | 1 | 3 | 3 | 1 | 1 | 3 | 2 | 3 | 1 | 1 | 1 | 1 | 3 | 3 | 3 | 1 |
| **Domain 2: Patient/Participant selection** | | | | | | | | | | | | | | | | | | | | | | | | | | | | |
|  | Adambounou 2014 | Adhikari 2014 | Alkadi 2009 | Biegler 2013 | Blaivas 2009 | Boniface 2011 | Courreges 2005 | Dyer 2008 | Ito 2013 | Johnson 1998 | Kim 2015 | Kim 2016 | Kolbe 2015 | Kwon 2007 | Lee 2016 | Levine 2015 | Levine 2016 | Litelpo 2010 | Litelpo 2011 | Macedonia 2008 | McBeth 2011 | McBeth 2013 | Mikulik 2005 | Nikolic 2006 | Sibert 2008 | Song 2013 | Strode 2003 | Zennaro 2016 |
| 1. *Unwell patients?* | 2 | 3 | 1 | 1 | 1 | 3 | 2 | 1 | 3 | 1 | 1 | 1 | 1 | 3 | 3 | 3 | 3 | 2 | 2 | 2 | 3 | 3 | 1 | 3 | 3 | 3 | 3 | 1 |
| 1. *Appropriate simulation?* | 4 | 3 | 4 | 4 | 4 | 2 | 4 | 4 | 2 | 4 | 4 | 4 | 4 | 1 | 2 | 2 | 2 | 2 | 2 | 2 | 3 | 2 | 2 | 2 | 3 | 2 | 1 | 4 |
| 1. *Level of expertise described?* | 3 | 1 | 1 | 1 | 1 | 1 | 3 | 1 | 1 | 2 | 1 | 1 | 1 | 2 | 1 | 1 | 1 | 1 | 1 | 3 | 1 | 1 | 1 | 2 | 3 | 1 | 1 | 1 |
| 1. *Methods of participation?* | 2 | 2 | 1 | 3 | 1 | 2 | 2 | 1 | 2 | 1 | 1 | 1 | 2 | 2 | 2 | 2 | 2 | 2 | 2 | 1 | 2 | 2 | 2 | 2 | 2 | 3 | 2 | 1 |
| 1. *Innaproprite exclusiosn?* | 2 | 2 | 2 | 2 | 1 | 2 | 2 | 1 | 2 | 1 | 1 | 1 | 2 | 2 | 3 | 3 | 3 | 3 | 2 | 2 | 3 | 2 | 2 | 2 | 2 | 3 | 2 | 1 |
| 1. ***Selection of participants led to bias?*** | 2 | 3 | 2 | 2 | 1 | 3 | 2 | 1 | 2 | 1 | 1 | 1 | 2 | 2 | 2 | 2 | 3 | 3 | 3 | 2 | 3 | 2 | 3 | 2 | 3 | 3 | 2 | 1 |
| **Domain 3: Index test(s)** | | | | | | | | | | | | | | | | | | | | | | | | | | | | |
|  | Adambounou 2014 | Adhikari 2014 | Alkadi 2009 | Biegler 2013 | Blaivas 2009 | Boniface 2011 | Courreges 2005 | Dyer 2008 | Ito 2013 | Johnson 1998 | Kim 2015 | Kim 2016 | Kolbe 2015 | Kwon 2007 | Lee 2016 | Levine 2015 | Levine 2016 | Litelpo 2010 | Litelpo 2011 | Macedonia 2008 | McBeth 2011 | McBeth 2013 | Mikulik 2005 | Nikolic 2006 | Sibert 2008 | Song 2013 | Strode 2003 | Zennaro 2016 |
| 1. *Reviewers blinded?* | 4 | 2 | 4 | 1 | 1 | 3 | 2 | 1 | 4 | 3 | 2 | 1 | 2 | 3 | 1 | 1 | 1 | 2 | 1 | 3 | 3 | 1 | 1 | 1 | 2 | 3 | 1 | 1 |
| 1. *Appropriate measure?* | 3 | 2 | 2 | 1 | 3 | 2 | 3 | 1 | 3 | 2 | 1 | 2 | 1 | 3 | 1 | 1 | 1 | 3 | 3 | 3 | 3 | 3 | 2 | 2 | 3 | 1 | 1 | 1 |
| 1. *Appropriate measured of subjective outcome?* | 3 | 1 | 3 | 4 | 1 | 1 | 1 | 1 | 3 | 1 | 1 | 1 | 3 | 3 | 1 | 1 | 1 | 1 | 1 | 3 | 3 | 3 | 1 | 4 | 1 | 1 | 1 | 1 |
| 1. *Appropriate statistical tests?* | 4 | 1 | 1 | 1 | 1 | 1 | 1 | 1 | 1 | 4 | 1 | 1 | 1 | 4 | 1 | 3 | 3 | 4 | 3 | 1 | 4 | 1 | 3 | 4 | 1 | 1 | 1 | 1 |
| 1. *Confidence intervals?* | 4 | 1 | 4 | 1 | 3 | 3 | 3 | 4 | 1 | 4 | 1 | 1 | 1 | 3 | 1 | 1 | 1 | 4 | 3 | 4 | 4 | 3 | 4 | 4 | 3 | 1 | 1 | 1 |
| 1. *Conduct or interpretation of index test caused bias?* | 2 | 3 | 2 | 1 | 2 | 3 | 3 | 1 | 3 | 2 | 2 | 1 | 1 | 3 | 1 | 1 | 1 | 3 | 3 | 3 | 3 | 2 | 2 | 2 | 2 | 2 | 1 | 1 |
| **Domain 4: Reference standard** | | | | | | | | | | | | | | | | | | | | | | | | | | | | |
|  | Adambounou 2014 | Adhikari 2014 | Alkadi 2009 | Biegler 2013 | Blaivas 2009 | Boniface 2011 | Courreges 2005 | Dyer 2008 | Ito 2013 | Johnson 1998 | Kim 2015 | Kim 2016 | Kolbe 2015 | Kwon 2007 | Lee 2016 | Levine 2015 | Levine 2016 | Litelpo 2010 | Litelpo 2011 | Macedonia 2008 | McBeth 2011 | McBeth 2013 | Mikulik 2005 | Nikolic 2006 | Sibert 2008 | Song 2013 | Strode 2003 | Zennaro 2016 |
| 1. *Reference standard?* | 3 | 1 | 4 | 1 | 1 | 3 | 1 | 3 | 3 | 1 | 1 | 1 | 3 | 3 | 1 | 1 | 1 | 3 | 1 | 1 | 3 | 4 | 1 | 1 | 3 | 1 | 1 | 1 |
| 1. *Reference standard appropriate?* | 4 | 2 | 4 | 1 | 2 | 4 | 1 | 4 | 4 | 1 | 1 | 1 | 4 | 4 | 1 | 1 | 3 | 4 | 1 | 1 | 4 | 4 | 2 | 1 | 4 | 1 | 1 | 1 |
| 1. *Reference standard interpreted blindly?* | 4 | 1 | 4 | 1 | 1 | 4 | 2 | 4 | 4 | 1 | 1 | 1 | 4 | 4 | 1 | 1 | 1 | 4 | 1 | 3 | 4 | 4 | 1 | 1 | 4 | 3 | 1 | 1 |
| 1. *Could the reference standard introduced bias?* | 4 | 3 | 4 | 1 | 2 | 4 | 2 | 4 | 4 | 2 | 1 | 1 | 4 | 4 | 1 | 1 | 2 | 4 | 1 | 3 | 4 | 4 | 2 | 1 | 4 | 2 | 1 | 1 |
| **Domain 5: Flow and timing** | | | | | | | | | | | | | | | | | | | | | | | | | | | | |
|  | Adambounou 2014 | Adhikari 2014 | Alkadi 2009 | Biegler 2013 | Blaivas 2009 | Boniface 2011 | Courreges 2005 | Dyer 2008 | Ito 2013 | Johnson 1998 | Kim 2015 | Kim 2016 | Kolbe 2015 | Kwon 2007 | Lee 2016 | Levine 2015 | Levine 2016 | Litelpo 2010 | Litelpo 2011 | Macedonia 2008 | McBeth 2011 | McBeth 2013 | Mikulik 2005 | Nikolic 2006 | Sibert 2008 | Song 2013 | Strode 2003 | Zennaro 2016 |
| 1. *Was there an appropriate interval before reference standard?* | 4 | 1 | 4 | 1 | 1 | 4 | 2 | 4 | 4 | 4 | 1 | 1 | 4 | 4 | 1 | 1 | 1 | 4 | 1 | 3 | 4 | 4 | 1 | 1 | 4 | 1 | 1 | 1 |
| 1. *If teaching outcomes assessment/ measurement?* | 4 | 4 | 4 | 4 | 4 | 3 | 4 | 4 | 4 | 4 | 1 | 4 | 1 | 3 | 4 | 2 | 2 | 4 | 4 | 4 | 4 | 4 | 4 | 3 | 4 | 4 | 4 | 4 |
| 1. *Did all patients receive a reference standard?* | 4 | 1 | 4 | 1 | 1 | 4 | 1 | 4 | 4 | 3 | 1 | 1 | 4 | 3 | 1 | 1 | 1 | 4 | 1 | 1 | 3 | 4 | 1 | 1 | 4 | 1 | 2 | 1 |
| 1. *Did patients receive the same reference standard?* | 4 | 1 | 4 | 1 | 1 | 4 | 1 | 4 | 4 | 3 | 3 | 1 | 4 | 3 | 1 | 1 | 1 | 4 | 1 | 1 | 3 | 4 | 1 | 1 | 4 | 1 | 2 | 1 |
| 1. *Were all patients/participants included in the analysis?* | 2 | 1 | 2 | 1 | 1 | 2 | 2 | 3 | 2 | 3 | 1 | 1 | 1 | 2 | 1 | 1 | 1 | 2 | 1 | 2 | 2 | 1 | 1 | 1 | 4 | 1 | 2 | 1 |
| 1. *Could the patient/participant flow have introduced bias?* | 2 | 2 | 2 | 1 | 1 | 3 | 2 | 2 | 2 | 3 | 1 | 1 | 1 | 2 | 1 | 1 | 1 | 2 | 1 | 2 | 2 | 1 | 1 | 1 | 4 | 1 | 2 | 1 |
| **Domain 6: Telemedicine/ Feasibility specific concerns** | | | | | | | | | | | | | | | | | | | | | | | | | | | | |
|  | Adambounou 2014 | Adhikari 2014 | Alkadi 2009 | Biegler 2013 | Blaivas 2009 | Boniface 2011 | Courreges 2005 | Dyer 2008 | Ito 2013 | Johnson 1998 | Kim 2015 | Kim 2016 | Kolbe 2015 | Kwon 2007 | Lee 2016 | Levine 2015 | Levine 2016 | Litelpo 2010 | Litelpo 2011 | Macedonia 2008 | McBeth 2011 | McBeth 2013 | Mikulik 2005 | Nikolic 2006 | Sibert 2008 | Song 2013 | Strode 2003 | Zennaro 2016 |
| 1. *Security measures used?* | 1 | 3 | 3 | 1 | 1 | 3 | 1 | 3 | 3 | 3 | 1 | 1 | 3 | 3 | 3 | 3 | 3 | 1 | 1 | 3 | 3 | 3 | 3 | 3 | 3 | 3 | 3 | 1 |
| 1. *Receiving or sending environments typical?* | 1 | 2 | 1 | 1 | 1 | 3 | 1 | 1 | 2 | 1 | 3 | 3 | 1 | 3 | 2 | 1 | 2 | 2 | 2 | 1 | 1 | 1 | 3 | 3 | 1 | 1 | 1 | 1 |
| 1. *Technical standards reported?* | 1 | 3 | 4 | 3 | 1 | 3 | 1 | 1 | 2 | 1 | 1 | 1 | 3 | 3 | 1 | 3 | 1 | 1 | 1 | 1 | 1 | 1 | 1 | 3 | 1 | 3 | 1 | 1 |
| 1. *Was the level of cost described?* | 1 | 3 | 4 | 3 | 1 | 3 | 3 | 3 | 3 | 3 | 3 | 3 | 3 | 3 | 3 | 3 | 1 | 3 | 3 | 3 | 3 | 3 | 3 | 1 | 3 | 3 | 3 | 1 |
| 1. *Were technical barriers described?* | 1 | 1 | 3 | 1 | 1 | 1 | 1 | 1 | 1 | 3 | 1 | 3 | 1 | 3 | 1 | 1 | 1 | 1 | 1 | 1 | 3 | 3 | 3 | 3 | 1 | 1 | 1 | 1 |
| **Domain 7: Concerns regarding applicability** | | | | | | | | | | | | | | | | | | | | | | | | | | | | |
|  | Adambounou 2014 | Adhikari 2014 | Alkadi 2009 | Biegler 2013 | Blaivas 2009 | Boniface 2011 | Courreges 2005 | Dyer 2008 | Ito 2013 | Johnson 1998 | Kim 2015 | Kim 2016 | Kolbe 2015 | Kwon 2007 | Lee 2016 | Levine 2015 | Levine 2016 | Litelpo 2010 | Litelpo 2011 | Macedonia 2008 | McBeth 2011 | McBeth 2013 | Mikulik 2005 | Nikolic 2006 | Sibert 2008 | Song 2013 | Strode 2003 | Zennaro 2016 |
| 1. *Reference standard applicable?* | 4 | 3 | 4 | 1 | 3 | 4 | 1 | 4 | 1 | 1 | 1 | 1 | 1 | 4 | 1 | 1 | 1 | 1 | 1 | 4 | 4 | 4 | 2 | 4 | 4 | 1 | 1 | 1 |
| 1. *Included patients applicable?* | 2 | 3 | 1 | 2 | 1 | 3 | 2 | 1 | 3 | 2 | 1 | 1 | 1 | 1 | 1 | 1 | 1 | 3 | 2 | 2 | 3 | 3 | 2 | 2 | 3 | 3 | 2 | 1 |
| 1. *Conduct of ultrasound applicable?* | 1 | 3 | 1 | 2 | 3 | 1 | 1 | 1 | 2 | 3 | 1 | 1 | 1 | 1 | 1 | 1 | 1 | 1 | 1 | 1 | 1 | 1 | 1 | 1 | 1 | 1 | 1 | 1 |
| 1. *Mode of telecommunications applicable?* | 1 | 1 | 1 | 1 | 3 | 3 | 1 | 1 | 1 | 3 | 1 | 1 | 1 | 1 | 1 | 1 | 1 | 1 | 1 | 1 | 1 | 1 | 1 | 1 | 1 | 1 | 1 | 1 |
