# Supplementary material for: Telesonography in emergency medicine: A systematic review
Source: PLoS One. 2018 May 3;13(5):e0194840. doi: 10.1371/journal.pone.0194840 (PMC5933714; doi:10.1371/journal.pone.0194840)
Supplement: S6 Table — (DOCX) [file pone.0194840.s007.docx]

# **S6 Table: Technical Feasibility**

| **Study ID** | **Success rate** | **Image quality** | **Time taken** | **Problems/notes** |
| --- | --- | --- | --- | --- |
| **Adambounou 2014** | NR | NR | **Transmission time:**  1.5s [synchronous]  3 mins [asynchronous transmission of 3D files] | Transmission possible even using low bandwidth 512kbps, although distortion of colour images with lower bandwidth, 256 kbps. |
| **Adhikari 2014** | **Image Transmission:** 100% successful | **Image quality:**  Scale: Likert [1 -10]  Grand mean: 7.5 | **Transmission time:**  Median: 1min 22.5s | No transmission losses. |
| **Biegler 2013** | **Image Transmission:** 100% successful | NR | NR | Issues with connection reliability and troubleshooting of the off the shelf components. |
| **Blaivas 2004** | **Identification of Structures:**  Inter-technology agreement with mean kappa value=0.64 | **Image quality:**  Scale: Likert [1 -10]  Phone - 7.85, Thermal: 7.65  **Diagnostic Confidence in Images:**  Scale: Likert [1 -10]  Phone - Mean 6.9, Thermal - Mean 7.9 | **Transmission time:**  Median: 1 min 38s [for single still image] | Measurements were too small to read using the camera phones. |
| **Boniface 2011** | **Image Transmission:** 100% successful | NR | **Examination time:**  Median: 4 min 22s | Communication problems concerning terms used to instruct novices. |
| **Courreges 2005** | **Image Transmission:** 78% successful | **Image quality:**  Scale: Not Specified  25% of cases scans of low resolution | **Examination time:**  Mean: 24 min | Disagreements with the final diagnosis occurred with lesions < 0.4 -3.8cm in size. |
| **Dyer 2008** | **Identification of Structures:**  98% FAST and 100% EFAST key structures seen  **Clinical Utility:** telesonography enabled confirmation of diagnosis in 35% cases | NR | NR | NR |
| **Ito 2013** | NR | NR | **Examination time:**  Per 1 FAST area mean 5 mins 54s  Whole examination complete within 9 mins | The grand mean of attachment time to all 4 FAST areas 221.7s The attachment time for overweight examinee was significantly longer than slender or normal build. |
| **Johnson 1998** | **Clinical Utility:** Scanning prevented transfer in 42% of all cases and influenced management in 59% of all cases | **Image quality:**  Scale: Not Specified  No problems with image degradation or quality | NR  **Transmission time:**  5 mins | Significant proportion of patients lost to follow up. |
| **Kim 2015** | **Identification of Structures:**  Appendix identification not possible or uncertain in 11.3% of examinations | **Image quality:**  Scale: Likert [1-5]  Mean: 4.18 | NR | NR |
| **Kim 2016** | NR | **Image quality:**  Scale: Likert [1 – 5]  Mean [Ultrasound]: 4.53 | NR | NR |
| **Kwon 2015** | **Image Transmission**: 100% successful | **Image quality:**  Scale: Not Specified  All downloaded DICOMs and video-streams adequate for interpretation. | NR | NR |
| **Kolbe 2015** | **New Diagnosis:**  52.3% of cases  **Clinical Utility:** Change in management in 48% of cases | **Image quality:**  Scale: Not Specified  All real-time video stream and still capture images were considered to be diagnostic quality. | NR | NR |
| **Lee 2016** | **Identification of structures:** 87.8 % of appendixes seen in telementored scans vs 91.1 % of reference [onsite mentored] scans | **Image quality:**  Scale: 1-5  Mean: 4.5 | **Examination time:**  4 mins 51 [telementored] 4 mins 2.9 s [onsite mentor] | NR |
| **Levine 2015** | NR | **Image Quality:**  Scale: 1-5  Grand mean: 4.5  **Diagnostic Confidence in Images:**  Scale: % of reviewers who considered images clinically useful  Percentage of Reviewers: 96% | **Examination time:**  Mean: 5 mins 48s | NR |
| **Levine 2016** | NR | **Image Quality:**  Scale: 1-5  Facetime Grand mean= 4.6  **Diagnostic Confidence in Images:**  Scale: 1-5  Facetime Grand mean: 4.0  Conventional images: Grand mean 4.2 | NR | NR |
| **Litelpo 2010** | **Identification of structures:**  100% relevant anatomy seen | **Diagnostic Confidence in Images:**  Scale: % clips with adequate frame rate  3G: 50% clips inadequate  Wi-Fi: 12.5% clips inadequate | **Transmission time:**  2.7 s | Lung sliding, internal jugular views and cardiac views frame rate inadequate. Frame rate dependent on the depth and ultrasound machine ranged from 24.8 fps at 4.7 cm to 5.8 fps at depth of 30 cm. |
| **Litelpo 2011** | **Image Transmission**:  Skype Wi-Fi: 100% successful  Skype 3G: 100% successful  iChat Wi-Fi: 100% successful  IChat 3G: 0% successful | **Image quality:**  Scale: % Image quality compared to original using Likert 1-10 scale  Skype Wi-Fi - 82%, Chat Wi-Fi - 91%, Skype 3G - 57%  **Diagnostic Confidence in Images:**  Scale: % of reviewers’ judgements which rated image quality as sufficient to make diagnosis  Skype Wi-Fi - 90%, Chat Wi-Fi - 100%, Skype 3G - 75%, IChat 3G - NA  **TOTAL:** 91.3% sufficient quality. | NR | iChat was unable to establish a connection through the 3G network, owing to an error of lack of sufficient bandwidth required to initiate a video connection. |
| **Macedonia 1998** | NR | **Image quality**:  Scale: Not specified. “Blur, resolution, sharpness, contrast sensitivity and image detail remained the same as conventional scan.”  **Diagnostic Confidence in Images:**  Scale: Not specified. “Diagnostic quality was unchanged from a conventional scan.” | **Transmission time:**  1-228 minutes | Image artefacts due to 3G technique, e.g. Posterior shadowing from fluid filled structures which gave 3D images unusual appearance. |
| **McBeth 2011** | **Identification of structures:**  Lung sliding viewed in all 8 interactions. | **Image quality:**  Scale: Not specified  Excellent in all scans. | NR | NR |
| **McBeth 2013** |  | **Image quality:**  Scale: 1-5  Grand mean: 4.21 | NR | NR |
| **Mikulik 2016** | **Identification of structures:**  100% Middle cerebral arteries, 100% Inferior cerebral arteries, 44% Anterior cerebral arteries,  88% Basilar arteries. | NR | **Examination time:**  Carotid Doppler: 30 mins [telementored] 15 mins [in Person expert]  Transcranial scan  45 mins [telementored] 18 mins [in person expert] | Variable insonation rates and number of suboptimal insonation windows. |
| **Nikolic 2006** | **Identification of structures:**  73.3% organs visible on captured images | NR | NR | NR |
| **Sibert 2008** | NR | **Image quality:**  Scale: 1–9  Stills: 4.0  Movies: 2.7 | NR | Reduction in resolution from the uncompressed baseline images, Slow frame rates, Loss of signal and slow transmission intervals. |
| **Strode 2003** | **Transmission success:**  Microwave Vest: 100%  VSAT satellite: 100%  INMARSAT satellite: 100%  Lifelink: 86% | **Image quality:**  Scale: 1-5  Microwave Vest - 3.2, VSAT satellite - 3.0, INMARSAT satellite - 1.3, Lifelink - 2.0, Total - 3.0 | NR | Use of WAN system, Lifelink system loss of signal 3s (max.75 s). Longer losses >5s associated maintenance 3 mins 39 s lost of total 24 mins video |
| **Zennaro 2016** | NR | **Image quality:**  Scale: % category  Poor - 0.0%, Fair - 7.7%, Good - 15.4%,  Very good - 42.3%, Excellent - 34.6% | **Examination time:**  Mean: 6mins 30s | Technical difficulties 2 [3.8%] cases: 1 x network failure 1x unable set up communication. |
